# Patient-reported experiences and patient-reported experience measures in primary care, home care, and community settings. A scoping review

**DOI:** 10.3389/fresc.2026.1966101

**Published:** 2026-09-24

**Authors:** Ilaria Marcomini, Nicoletta Troiano, Mattia Bozzetti, Debora Rosa, Giulia Villa, Andrea Poliani, Duilio Fiorenzo Manara

**Affiliations:** 1 Center for Nursing Research and Innovation, Vita-Salute San Raffaele University, Milan, Italy; 2 Otorhinolaryngology, Surgical Department, IRCCS Fondazione Istituto Nazionale dei Tumori, Milan, Italy; 3 Direction of Health Professions, ASST Cremona, Cremona, Italy

**Keywords:** community, patient reported experience, patient reported experience measures, primary care, review

## Abstract

**Introduction:**

Patient-reported experience measures (PREMs) are increasingly used to evaluate person-centred and value-based healthcare. However, evidence regarding their application in primary, home, and community care remains fragmented.

**Objective:**

To map how patient-reported experiences (PREs) and patient-reported experience measures (PREMs) have been conceptualized, measured, and used across primary, home, and community care settings.

**Methods:**

The review followed Joanna Briggs Institute methodology and PRISMA-ScR reporting guidelines. MEDLINE, CINAHL, PsycINFO, Scopus, and Web of Science were searched from inception to July 2025. Peer-reviewed studies investigating PREs or PREMs in primary, home, or community care were included. Data were synthesized using descriptive statistics, thematic content analysis, and narrative synthesis.

**Results:**

Overall, 168 studies were included, predominantly quantitative and conducted in high-income countries. Among them, 105 exclusively used validated PREMs, 45 used *ad hoc* instruments, and 14 combined both approaches. The most frequently assessed domains were communication and information, access to services, patient–professional relationships, quality of care, continuity, care coordination, and patient involvement. Equity-related domains were less consistently represented, while pediatric populations, older adults, and low- and middle-income countries were underrepresented.

**Discussion:**

PREM use in primary, home, and community care is increasing but remains heterogeneous in terms of conceptualization, measurement approaches, and domains assessed. Greater standardization of PREMs and broader inclusion of equity-sensitive domains are needed to strengthen the relevance and comparability of patient experience assessment across care settings.

**Conclusion:**

PREMs offer substantial potential to support person-centred quality improvement beyond hospital settings, but their current use does not yet fully capture the diversity of patient experiences. Future research should prioritize standardized and context-sensitive measurement approaches, greater attention to equity, and stronger representation of underserved populations and low- and middle-income settings.

**Systematic Review Registration:**

https://doi.org/10.17605/OSF.IO/R5SQF, Identifier: 10.17605/OSF.IO/R5SQF.

## 1 Introduction

Value-based healthcare (VBHC) has progressively shifted the focus of health systems from the volume of services delivered to the value generated for patients, commonly understood as the relationship between outcomes that matter to people and the resources required to achieve them (1). Although VBHC has often been operationalized within hospital-based pathways, its principles are increasingly relevant to primary care, home care, and community services. In these settings, population ageing, multimorbidity, chronic illness, and social vulnerability require models of care that are longitudinal, coordinated, accessible, and person-centred (2–4). Accordingly, in these settings, value cannot be adequately assessed through clinical outcomes, service utilization, or cost-related indicators alone.

It also requires systematic attention to how people experience care, particularly in relation to accessibility, communication, continuity, coordination, and the capacity of services to respond to patients' needs over time (4, 5).

Patient-reported experiences (PREs) provide direct accounts of what occurs during care from the patient's perspective. They include aspects such as access to services, clarity of information, communication with healthcare professionals, involvement in decision-making, emotional support, respect for individual preferences, coordination among providers, and continuity across care transitions (6–9). Patient-reported experience measures (PREMs) are measures that assess the experiences reported by patients regarding their healthcare services (7, 8, 10). Unlike satisfaction surveys, which may be influenced by expectations, gratitude, or subjective judgement, PREMs focus on observable and actionable aspects of the care process. For this reason, they are increasingly recognized as useful tools for quality improvement, accountability, benchmarking, and service redesign (7, 11, 12). PREMs also differ from patient-reported outcome measures (PROMs), which primarily assess patients' perceptions of health status, symptoms, functioning, or quality of life, whereas PREMs focus on experiences of care delivery and interactions with services (13, 14). To date, PREMs have been developed and implemented predominantly in hospital, inpatient, and condition-specific contexts, whereas their use in primary care, home care, and community services remains less clearly described and more fragmented (6, 8, 9). This represents a relevant knowledge gap, as these settings are central to the management of chronic conditions, long-term care needs, prevention, health promotion, and support for people living in situations of clinical or social vulnerability. They are also characterized by relational continuity, multidisciplinary collaboration, and frequent transitions across services. However, it remains unclear how patient experience is conceptualized and measured beyond hospital-based care, which populations and clinical conditions have been addressed, which experiential domains are most frequently captured, and whether available instruments are validated measures or tools developed for individual studies. This issue is particularly relevant for nursing. Nurses play a key role in primary, home, and community care by promoting accessibility, continuity, patient education, self-management support, shared decision-making, and coordination across services (15). These areas closely correspond to core domains explored through PREs and PREMs, including communication, involvement in decisions, coordination, and continuity of care (8, 16). A clearer understanding of how PREs and PREMs are used in these settings may therefore support healthcare professionals and policymakers in identifying unmet needs, integrating patient perspectives into care planning, and informing quality improvement initiatives within person-centred, integrated, and value-based models of care (7, 17).

This scoping review aimed to map how PREs and PREMs have been conceptualized, measured, and used in primary, home, and community care. Specifically, it sought to describe the populations and care contexts addressed, the experiential domains captured, the instruments used, and whether these instruments were validated PREMs or tools developed *ad hoc*.

## 2 Methods

### 2.1 Study design

A scoping review was conducted in accordance with the Joanna Briggs Institute (JBI) Manual for Evidence Synthesis (18) and reported according to the PRISMA Extension for Scoping Reviews (PRISMA-ScR) checklist (19). Before conducting the search, PROSPERO and the JBI Systematic Review Register were consulted to determine whether protocols or reviews addressing the same topic were already underway. The review protocol was preregistered on the Open Science Framework (DOI: 10.17605/OSF.IO/R5SQF).

### 2.2 Research question

The review was guided by the following research question: “What patient-reported experiences are described, and how are they measured, across different models of care delivery in primary care, home care and community care settings?” The question was subsequently structured using the Population–Concept–Context (PCC) framework (18) as shown in Table 1.

**Table 1:** PCC framework structured according to the research question.

PCC element	Component	Description
Population	P	Individuals of all ages
Concept	C	Patient-reported experiences, defined as patients’ direct accounts of what they encounter, perceive, and experience during care, and patient-reported experience measures, defined as standardized or structured instruments used to collect these experiences systematically.
Context	C	Primary, home-based, and community healthcare settings delivered outside hospital inpatient care, including integrated and transitional care pathways when focused on community-based service delivery.

### 2.3 Eligibility criteria and selection of sources of evidence

We included peer-reviewed publications written in English, without date, geographical, or methodological restrictions. Quantitative, qualitative, mixed-methods, observational, experimental, quasi-experimental, implementation studies and reviews were considered if they described PREs, used validated or non-validated PREMs, or reported domains of patient experience such as communication, access, continuity, involvement, coordination, or support. Studies not addressing PREs/PREMs, unavailable in full text, and non-peer-reviewed sources were excluded. Grey literature was excluded because the review aimed to map evidence from peer-reviewed, indexed scientific literature to ensure methodological rigor, transparency, and reproducibility.

### 2.4 Search strategy

A three-step search strategy process was applied following the JBI guidelines (20). An initial search in MEDLINE via PubMed and CINAHL was conducted to identify the main keywords describing the concept. The defined strategy was developed in collaboration with and subsequently peer-reviewed by an expert health sciences librarian to ensure methodological accuracy. It was then searched in MEDLINE, CINAHL, PsycINFO, Scopus, and Web of Science. The final search strategies are reported in the Supplementary File 1. Searches were updated on July 15–16, 2025, and backward citation searches were conducted. Records were managed in Rayyan, and screening was performed independently by two reviewers (N.T., A.P.) in two stages: title/abstract and full-text assessment (21). Disagreements were resolved by consensus between the two independent reviewers and with the intervention of a senior reviewer (I.M.). The entire selection process was reported using the PRISMA 2020 flow diagram (22).

### 2.5 Data extraction

Data extraction was undertaken through a systematic and iterative process. A preliminary extraction form was developed during protocol preparation and refined throughout the review. Extracted data included metadata, study aims, population characteristics, methodology, context, setting, model of care, reported PREs, PREMs, and key findings (Tables 2, 3). The form was pilot-tested on a limited number of sources to ensure clarity, consistency, and relevance before full data extraction.

**Table 2:** Validated PREMs used across clinical conditions and their main domains.

Clinical condition	PREM instrument	Main domains
Multiple chronic conditions	Press Ganey Outpatient Medical Practice Survey	Communication; Patient–professional relationship; Time spent with the patient; Patient attention
	Patient Assessment of Chronic Illness Care (PACIC)	Treatment burden; Self-management and health management; Impact on daily life; Financial aspects; Access to services; Side effects; Care support and coordination
	Person-Centred Coordinated Care Experience Questionnaire (P3CEQ)	Communication and relationship; Patient involvement; Continuity of care; Care coordination; Care planning; Self-management
	Primary Care Assessment Tool—Short Form (PCAT-S)	Continuity of care; Coordination; Access; Comprehensiveness of care; Health perception
	Patient Assessment of Chronic Illness Care—Short Form (PACIC-11); CollaboRATE; Patient Activation Measure (PAM)	Self-management; Continuity of care; Care coordination; Patient involvement; Team support; Care organization
	Patient-Centered Coordination by a Care Team (PCCCT)	Care coordination and continuity; Roles and responsibilities; Patient involvement; Communication and relationship
Cardiovascular diseases	Nijmegen Continuity Questionnaire (NCQ)	Continuity of care; Communication and relationship; Care coordination
Respiratory diseases	Chronic Care Patient Reported Experience Measure (PREM-CC)	Communication and relationship; Support; Patient involvement; Perceived quality of care; Care coordination
Metabolic diseases	Patient Experience of Diabetes Service Survey (PEDS); Diabetes Reported Experience Measure Survey (DREMS); Inpatient Diabetes Patient Reported Experience Measure (Inpatient Diabetes PREM); EDP Diabetes Patient Experience Questionnaire (EDP Questionnaire); Outpatient Clinic Patient Reported Experience Measure (Outpatient Clinic PREM); Adolescent Patient Experience Questionnaire for Diabetes Care (APEQ-DC); Swedish National Diabetes Register Patient Experience Survey (NDR PREM); Type 1 Diabetes Youth Patient Reported Experience Measure (T1D Youth PREM)	Care planning; Self-management; Communication and relationship; Quality of care; Care transitions
Gastrointestinal diseases	Inventory to Assess Patient Satisfaction (IAPS)	Service organization; Communication and relationship; Perceived quality of care
General medical conditions	Patient-Reported Experiences and Outcomes of Safety in Primary Care (PREOS-PC)	Access; Service organization; Communication and relationship; Safety of care; Care coordination; Continuity of care; Emotional experience; Patient involvement
	Press Ganey Outpatient Medical Practice Survey	Access; Service organization; Communication and relationship; Patient involvement; Continuity of care; Care coordination; Perceived quality of care
Musculoskeletal diseases	European Task Force on Patient Evaluations of General Practice Care (EUROPEP)	Communication and relationship; Quality of care; Information; Care coordination; Continuity of care; Access
	Friends and Family Test (FFT)	Communication and relationship; Perceived quality of care; Access
Mental health disorders	EQUIP Physical Health PREM (EQUIP-PH-PREM)	Patient involvement; Communication and relationship; Quality of care; Self-management
	Patient Reported Experience Measure in Addiction Treatment (PREMAT-NL)	Self-management; Communication and relationship; Service organization; Environment; Care coordination; Continuity of care; Patient involvement
Oncological diseases	Ambulatory Oncology Patient Satisfaction Survey (AOPSS)	Communication and relationship; Environment; Access; Care coordination and continuity; Information; Emotional support
Neurological diseases	Epilepsy12 Patient/Carer and Child Experience Questionnaire (Epilepsy12 PREM)	Communication and relationship; Information; Patient involvement; Access; Service organization; Care coordination; Environment; Perceived quality of care
Renal diseases	Home Dialysis Care Experience (Home-DCE)	Communication and relationship; Support; Care coordination; Patient involvement; Perceived quality of care
Maternal health	Pregnancy and Maternity Care Patients’ Experiences Questionnaire (PreMaPEQ)	Communication and relationship; Patient involvement; Information; Care coordination; Service organization; Caregiver involvement; Emotional support; Environment; Continuity of care; Access
Hearing disorders	UK Audiology Care Questionnaire (CQI-based)	Environment; Communication and relationship; Quality of care; Service organization; Access; Patient involvement; Perceived quality of care

**Table 3:** Patient-Reported experience (PRE) categories, representative content, and frequency across the included studies.

PRE category (references)	Representative content	Frequency (n)
Communication and information	Communication with healthcare professionals; clear, complete and consistent information; health education; opportunity to ask questions; understanding of diagnosis, treatment and care pathway.	62
Access to services	Access to healthcare services; appointment availability; waiting times; accessibility; contact with healthcare providers; barriers to access.	51
Patient–professional relationship	Relationship with healthcare professionals; trust, empathy and respect; active listening; supportive and patient-centred care.	43
Quality of care	Perceived quality, effectiveness and benefits of healthcare services and treatments.	38
Service organization	Service organization; care pathways; care planning; coordination and continuity; treatment management; patient participation; service improvement.	34
Continuity of care	Continuity of care; care coordination; follow-up and monitoring; care transitions; integrated and ongoing care.	24
Emotional experience	Emotional and psychological impact; emotional support; anxiety, fear and frustration; coping; social impact.	23
Patient involvement	Patient involvement and participation; shared decision-making; patient empowerment and autonomy.	20
Care coordination	Care coordination; interprofessional collaboration; integrated and continuous care; information continuity.	18
Overall satisfaction	Overall satisfaction with care; willingness to recommend and reuse healthcare services.	16
Cultural safety	Cultural responsiveness and safety; respect for cultural values and preferences; cultural and language barriers; inclusion of social and cultural diversity.	12
Safety of care	Perceived safety of care; clinical and environmental safety; service reliability.	11
Symptom management	Symptom and pain management; symptom experience; disease control.	11
Social support	Peer support; group belonging; shared experiences; interpersonal learning.	11
Financial access to services	Financial burden; insurance coverage; transportation; service accessibility; social determinants of access to care.	9
Physical environment	Care environment; physical comfort; cleanliness and order.	8
Impact on daily life	Impact on daily functioning; work participation; quality of life.	7
Caregiver and family involvement	Caregiver and family involvement; family support.	6
Patient preferences	Care preferences; preferred care delivery; treatment preferences.	5
Privacy	Privacy; confidentiality of health information; respect for patient dignity.	4
Diagnostic testing experience	Physical discomfort; procedure-related discomfort; diagnostic test experience.	2
Care needs	Unmet healthcare needs; respect for patients’ values and needs.	2
Contextual appropriateness of services	Patient experience measurement; use of patient experiences for quality assessment.	1

### 2.6 Data analysis

Extracted data were synthesized using descriptive statistics, qualitative categorization, and narrative synthesis. Study characteristics, care settings, PREMs, domains, and models of care were summarized in tables, figures, and evidence maps. Patient-Reported Experiences were analyzed through descriptive content analysis and grouped into thematic categories reflecting patient experience domains and care contexts.

Individual PREs were examined and grouped into conceptually homogeneous categories based on their semantic affinity and substantive content. Different expressions referring to the same experiential construct were grouped within the same category, whereas distinct dimensions were kept separate when they described specific aspects of the patient experience. As the objective of this scoping review was to map the scope, characteristics, and use of PREs and PREMs across primary, home, and community care, rather than to evaluate the measurement properties of individual instruments or formulate recommendations for instrument selection, a formal COSMIN assessment was not conducted (23). Information on whether instruments were validated, adapted, or developed *ad hoc* was extracted and reported descriptively.

## 3 Results

### 3.1 Selection process of the included studies

A total of 494 records were identified through database searching (PubMed, *n* = 165; Web of Science, *n* = 91; PsycINFO, *n* = 42; and CINAHL, *n* = 41). After removing 202 duplicates, 292 records were screened by title and abstract, of which 46 were excluded. The full texts of the remaining 246 articles were assessed for eligibility. Following full-text review, 78 articles were excluded because they did not meet the inclusion criteria, primarily due to an inappropriate setting, lack of extractable PREs/PREMs data, exclusive focus on patient-reported outcomes (PROs/PROMs), non-peer-reviewed publication types, overlap with previously included studies, or unavailable full text. Overall, 168 studies were included in this scoping review (Figure 1).

**Figure 1:**
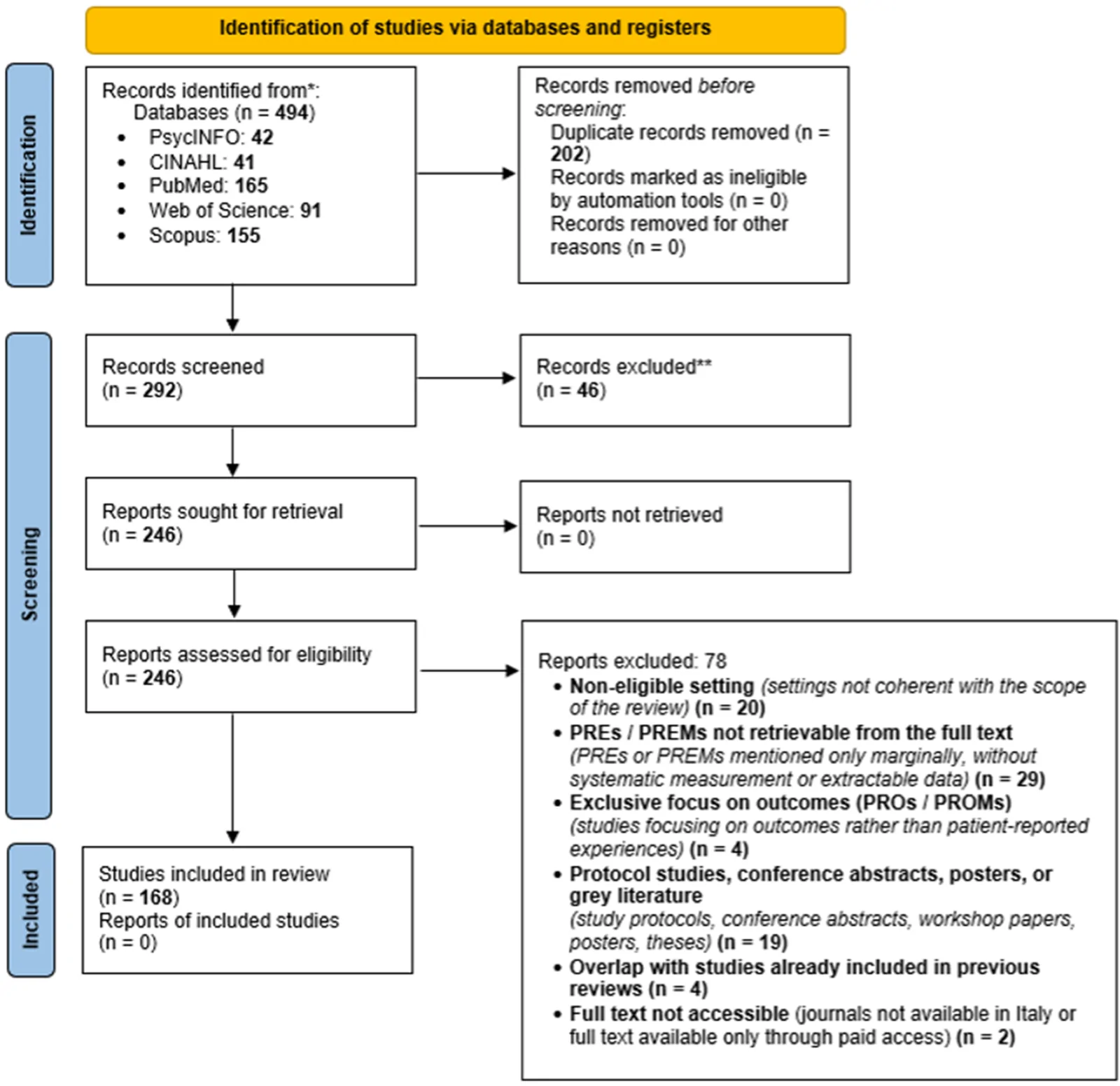
PRISMA flow diagram.

### 3.2 Characteristics of the included studies

A detailed description of the characteristics of the included studies and study populations is provided in Supplementary Table S2; therefore, individual study citations are omitted from these sections.

### 3.3 Study types, geographic distribution and publication timeline

The 168 studies were published between 2007 and July 2025. Overall, the review included 140 primary studies and 28 literature reviews. Quantitative designs (*n* = 100, 59.5%) predominated among the primary studies, followed by qualitative (*n* = 36, 21.4%) and mixed-methods (*n* = 32, 19.0%) designs. The included reviews (*n* = 28 16.7%) comprised systematic, scoping, narrative, rapid, and methodological reviews, thereby providing complementary evidence across different methodological approaches (Supplementary Table S2).

Figure 2 illustrates the temporal evolution of the evidence, the main publishing countries, trends across care settings and geographic regions, and the global distribution of study locations. Overall, the annual number of publications increased substantially after 2016, reaching its highest level in 2024 (*n* = 30) (Figure 2a). The evidence was generated predominantly in high-income countries, with the United Kingdom (*n* = 40), the United States (*n* = 26), and Canada (*n* = 19) contributing the largest number of studies (Figure 2b). Primary care was the most frequently investigated setting throughout the study period, although studies conducted in integrated or multiple care settings increased markedly in recent years (Figure 2c). Europe consistently contributed the largest number of publications over time, followed by North America, whereas Asia, Oceania, and other world regions remained comparatively underrepresented (Figure 2d). The geocoded distribution of the included studies is presented in Figures 2e–g.

**Figure 2:**
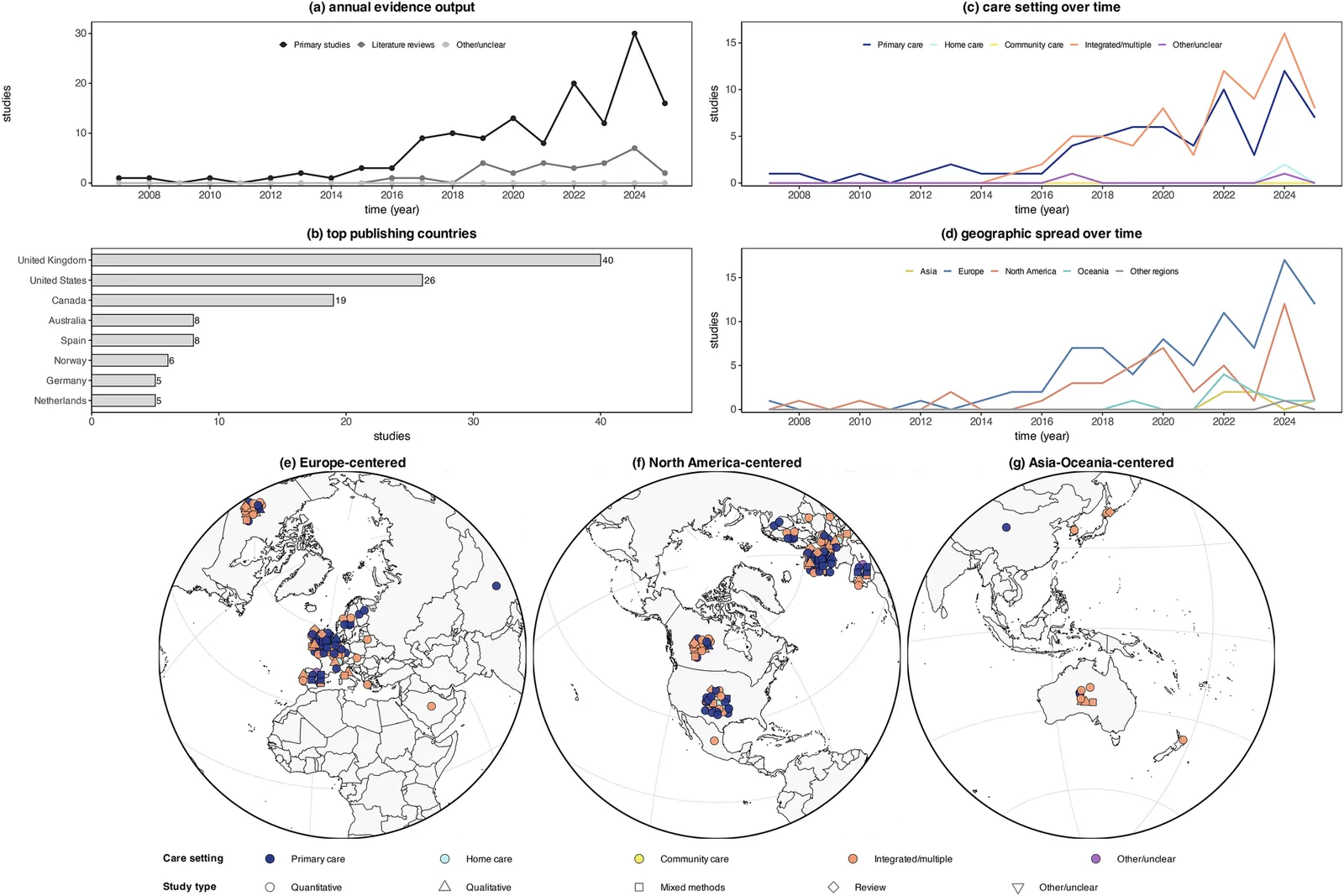
Panel **(a)** shows the annual number of studies by study type; panel **(b)** reports the countries contributing the largest number of studies; panel **(c)** shows annual trends by care setting; panel **(d)** shows annual trends by geographic region; and panels **(e–g)** display geocoded study locations using Europe-, North America-, and Asia–oceania-centred map projections. In the maps, point colour denotes care setting and point shape denotes study type.

### 3.4 Sample characteristics

Among the 140 primary studies, 79 (56.4%) focused on specific clinical conditions. The most frequently investigated populations were patients with multiple chronic conditions, followed by musculoskeletal disorders, mental health conditions, neurological and oncological diseases, and respiratory diseases. Cardiovascular conditions and other disease groups were less frequently represented, while a small proportion of studies focused on post-surgical or procedural care. The literature reviews included showed a similar pattern, with the greatest emphasis on mental health and substance use disorders and multiple chronic conditions, whereas oncology, neurology, nephrology, and other clinical areas were only occasionally addressed.

Adult populations predominated across both primary studies and literature reviews. Among primary studies, 109 (77.86%) focused on adults, whereas mixed-age samples accounted for 12 studies. Beyond age groups, few studies explicitly focused on populations experiencing social vulnerability, including socially vulnerable populations, veterans, multi-ethnic populations, individuals facing language barriers, people with low socioeconomic status, Indigenous populations, and frail older adults. When these dimensions were addressed, they were generally captured indirectly through domains such as access to services, financial access, cultural safety, social support, and contextual appropriateness of services, rather than through equity-specific PREMs or dedicated analytical frameworks. Pediatric and older adult populations were markedly underrepresented, with only four and three studies, respectively. Similarly, literature reviews mainly focused on adult populations, with fewer reviews including mixed-age samples or older adults.

### 3.5 Instruments used to assess patient-reported experiences

One-hundred and five studies exclusively used validated PREMs (12, 24–127) whereas 45 studies employed *ad hoc* instruments Fourteen studies adopted a combined approach using both validated PREMs and *ad hoc* instruments (128–172), Fourteen studies adopted a combined approach using both validated PREMs and *ad hoc* instruments (9, 16, 173–184), while only four studies did not explicitly report the instrument used.

The association between each included study and the PREM instruments is provided in Supplementary Table S2.

### 3.6 PREMs

Table 2 summarizes the main validated disease-specific PREMs identified in this review, indicating for each instrument its name, the principal domains assessed, and the clinical condition or patient population for which it was originally developed. The complete list of identified PREMs, the main domains that composed each PREM, the clinical condition, the population, and the age groups, together with their full bibliographic citations, are provided in Supplementary Table S3.

### 3.7 Categories of PRE identified through *ad hoc* instruments

The analysis of the categories of PREs identified in studies that exclusively used *ad hoc* instruments involved a process of thematic content categorization.

As shown in Table 3, the most frequently identified PRE categories were communication and information, access to services, patient–professional relationship, quality of care, and service organization. Less frequent but conceptually relevant categories included emotional experience, cultural safety, social support, patient preferences, caregiver involvement, privacy, and contextual appropriateness of services. The complete version of Table 3 with the full list of contributing studies and their bibliographic citations for each PRE category is available in Supplementary Table S4.

Figure 3 provides an overview of the distribution of PRE/PREM domains across out-of-hospital care settings, showing the frequency with which each experiential domain was investigated in primary care, home care, community care, and integrated or multiple care settings. Communication and information, access and affordability, patient–professional relationship, support and self-management, and quality of care were the most frequently represented domains across settings and were particularly prominent in primary care and integrated or multiple care settings. By contrast, home care and community care showed a narrower domain coverage, while equity and cultural safety, impact on daily life, and environment and privacy were less consistently represented across all settings.

**Figure 3:**
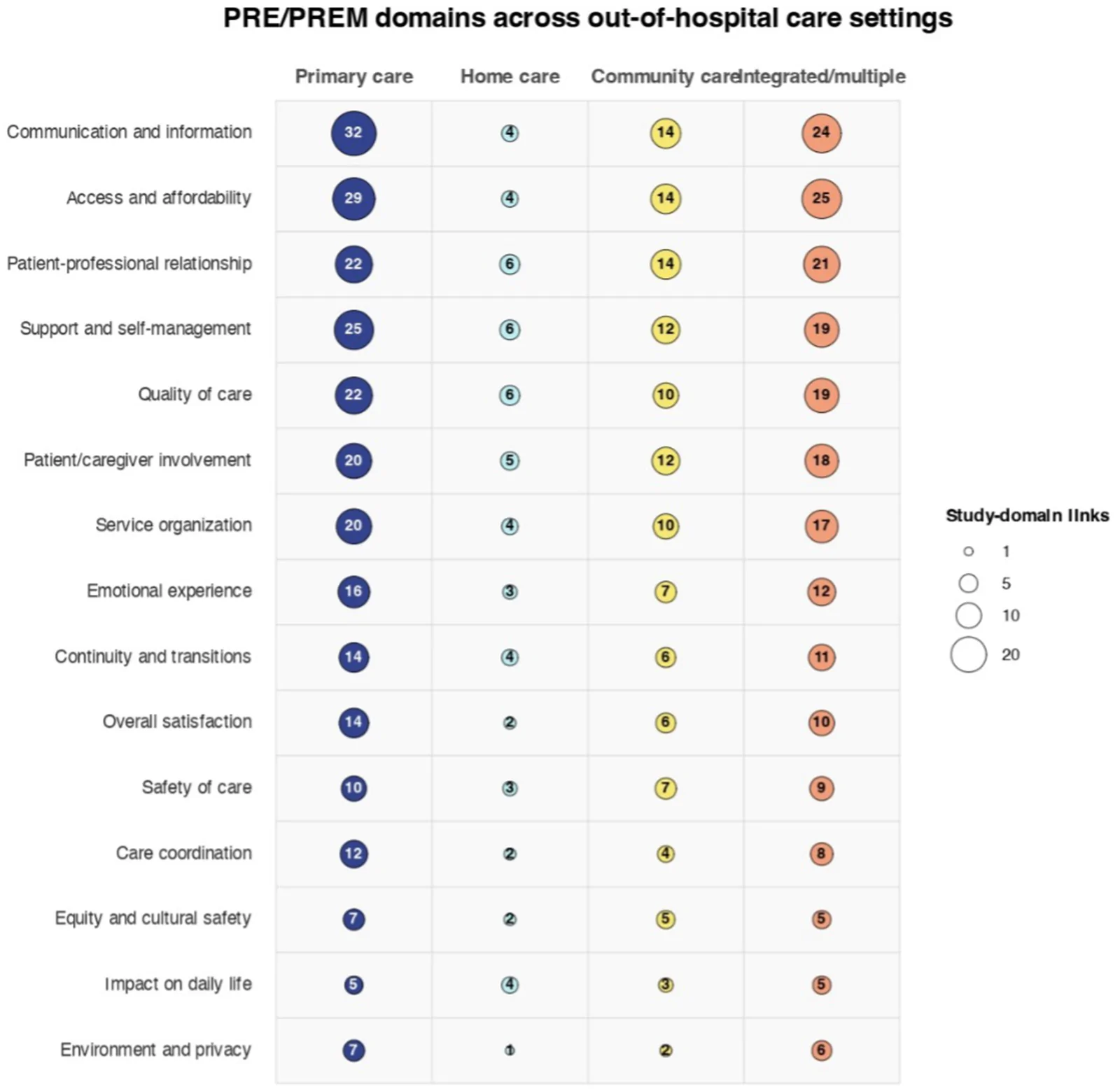
The bubble matrix displays the number of study–domain links for each experiential domain and care setting. Bubble size is proportional to the number of links, and numeric labels provide the exact counts. Domains are ordered by overall frequency across settings. Colours identify the care setting category: primary care, home care, community care, and integrated/multiple care. Because individual studies could address more than one experiential domain, values should be interpreted as domain occurrences across studies rather than mutually exclusive study counts.

## 4 Discussion

This scoping review systematically mapped PREs and PREMs across primary, home, and community care, examining their characteristics, patterns of use, and the main experiential domains assessed. The resulting evidence synthesis provides a practical resource for researchers, clinicians, healthcare managers, and policymakers by identifying the instruments currently available, the dimensions of patient experience most frequently assessed, and areas where evidence remains limited. These findings may support the selection and implementation of PREMs in community healthcare settings, promote greater consistency in patient experience measurement, and contribute to the development of person-centred, integrated, and value-based healthcare services.

The review identified a wide range of instruments applied across diverse clinical and organizational contexts. This diversity enabled patient experience to be explored from both quantitative and qualitative perspectives, reflecting the multidimensional nature of community-based care. This heterogeneity is informative in itself: it suggests that patient experience in community-based care is increasingly recognized as important, but that the field has not yet converged on a shared measurement architecture. As a result, comparisons across services, populations, and countries remain challenging.

The temporal distribution of publications showed an initial emergence of studies from 2007 onwards, followed by a marked increase after 2017. This trend reflects the growing interest in primary, home, and community care models developed to address the increasing burden of chronic diseases, multimorbidity, and population ageing, which have progressively challenged the ability of traditional hospital-centred models to meet contemporary healthcare needs (185, 186). Accordingly, community care has increasingly become the preferred setting for ensuring continuity of care, integrating health and social services, and reducing avoidable hospital admissions (187, 188). Consistent with this evolution, the literature also shows growing interest in the use of PREMs within community settings, driven by the need to support more continuous and person-centred care pathways that actively engage patients and caregivers in the care process (185, 186, 189).

The limited representation of low- and middle-income countries also raises an important equity issue. Primary, home, and community care services are often expected to reduce barriers to access and support people living with chronic illness, multimorbidity, disability, frailty, socioeconomic disadvantage, or social isolation (185, 188). However, the findings of this review suggest that the patient experience evidence base remains only partially equipped to capture these dimensions. Although several studies addressed domains that are closely related to equity, such as access to services, financial barriers, cultural safety, social support, patient preferences, caregiver involvement, and contextual appropriateness of care, these dimensions were less consistently represented than more established domains such as communication, access, and patient–professional relationships. This suggests that current PREMs may not fully capture the experiences of patients whose access to care and ability to navigate services are shaped by social, cultural, economic, or relational vulnerability.

With regard to population characteristics, most studies focused on adults, whereas pediatric populations were markedly underrepresented. This may reflect both the more limited availability of community-based pediatric services and the methodological challenges associated with measuring children's experiences, which often require caregiver involvement. Although the epidemiological transition toward chronic diseases and mental health disorders has increased the need for community- and home-based pediatric care (190), relatively few PREs and PREMs have been developed and validated for children, and those currently available remain less standardized than instruments designed for adults (191).

Another notable finding was the increasing use of PREMs in mental health and addiction services, where these instruments provide valuable insights into patients' experiences of therapeutic and rehabilitation pathways (192, 193). In these settings, PREMs may also contribute to reducing the stigma that continues to affect mental healthcare by supporting more person-centred, socially inclusive, and relationship-based models of care (194, 195).

An important finding of this review is the coexistence of validated PREMs and *ad hoc* questionnaires, confirming that the measurement of patient experience in community care remains an evolving field (10). Despite the diversity of instruments identified, the domains most frequently assessed were largely consistent and included communication, access to services, continuity of care, care coordination, patient–professional relationships, and patient involvement.

Conversely, *ad hoc* instruments appear to have been developed to address the specific needs of particular clinical settings, organizational contexts, or patient populations. Importantly, these instruments often allowed a more detailed exploration of dimensions that are less frequently captured by standardized PREMs, including emotional experience, cultural safety, social support, patient preferences, caregiver involvement, financial access, and the contextual appropriateness of services. These domains are particularly relevant from an equity perspective, as they may reveal barriers or unmet needs that remain invisible when patient experience is assessed only through generic measures of communication, access, or overall quality of care.

### 4.1 Implication for research and practice

The findings of this scoping review highlight the need for further research to improve the standardization and comparability of PREMs through the development and validation of instruments that can be applied across different community healthcare settings while remaining sufficiently flexible to accommodate the organizational, clinical, and cultural characteristics of diverse populations and care contexts.

A systematic evaluation of the measurement properties and methodological quality of the identified PREMs is also warranted. Future systematic reviews should apply the COSMIN methodology to assess properties such as content validity, structural validity, internal consistency, reliability, measurement error, construct validity, responsiveness, and cross-cultural validity or measurement invariance. Such evaluations would support the identification and selection of psychometrically sound and methodologically robust instruments. They would also clarify whether the available PREMs provide sufficiently reliable and valid data to inform research, quality improvement, and healthcare decision-making across primary, home, and community care settings.

Future studies should also address current evidence gaps by promoting research in underrepresented geographical regions and among populations that remain poorly investigated, including pediatric patients, older adults, people with multimorbidity or disability, migrants, ethnic or cultural minorities, individuals with low socioeconomic status, and those experiencing social isolation or limited health literacy. Particular attention should be paid to whether existing PREMs are sufficiently sensitive to detect inequities in experience of care, including barriers related to access, communication, affordability, cultural safety, caregiver involvement, and the contextual appropriateness of services. This requires strategies that balance standardization with contextual adaptation, ensuring that PREMs remain comparable across settings while being able to capture the experiences of populations at risk of being underserved.

Although the exploratory nature of this scoping review and the absence of a formal methodological quality assessment preclude prescriptive recommendations for clinical practice, the findings suggest that PREMs may represent valuable tools for monitoring the quality of primary, home, and community care services and for supporting quality improvement initiatives informed by the patient perspective. Their integration into routine practice could complement traditional clinical indicators by informing performance evaluation, service planning, and the assessment of integrated care models, while facilitating the identification of organizational weaknesses and unmet patient needs. From a policy perspective, the marked variability in the available instruments highlights the need for shared strategies to guide the selection, implementation, and routine use of PREMs, promoting a more systematic integration of patient experience into the evaluation and continuous improvement of community healthcare services.

### 4.2 Limitations

Several limitations should be acknowledged. First, the exclusion of grey literature, although intended to ensure methodological rigor, may have limited the identification of emerging evidence. In addition, the heterogeneity of definitions and terminology used across studies may have affected the classification of PRE domains and instruments, despite the use of an iterative categorization process. Finally, as expected in scoping reviews, no formal assessment of methodological quality, risk of bias, or measurement properties was performed. Accordingly, the COSMIN methodology was not applied, as the review was not designed as a systematic review of measurement properties or as a comparative appraisal of PREM quality. The findings should therefore be interpreted as a comprehensive mapping of the available evidence, instruments, and experiential domains rather than as an evaluation of the psychometric quality of individual PREMs.

## 5 Conclusion

This scoping review mapped the available evidence on PREs and PREMs in primary, home, and community care, highlighting the growing attention to the assessment of patient experience in community healthcare services.

Overall, the review confirms the increasing scientific interest in integrating the patient perspective into healthcare systems, while also highlighting important knowledge gaps. These include the fragmentation of measurement instruments, the limited standardization of PREMs, the underrepresentation of certain healthcare settings and populations, and the need for a better understanding of the contribution of PREs to improving the quality of community healthcare services.

The development of PREMs that are more standardized while remaining sufficiently flexible to accommodate different clinical, organizational, cultural, and socioeconomic contexts may facilitate more consistent national and international comparisons, while supporting service evaluation, healthcare planning, and the continuous quality improvement of primary, home, and community care. Greater attention to equity-sensitive domains may also help ensure that patient experience measurement captures the voices of populations whose needs are complex, underserved, or insufficiently represented in current evidence.

## Data Availability

The original contributions presented in the study are included in the article/Supplementary Material, further inquiries can be directed to the corresponding author/s.
