# Supplementary material for: Patient-reported experiences and patient-reported experience measures in primary care, home care, and community settings. A scoping review
Source: Front Rehabil Sci. 2026 Sep 24;7:1966101. doi: 10.3389/fresc.2026.1966101 (PMC13646105; doi:10.3389/fresc.2026.1966101)
Supplement: Supplementary file 1 [file Table1.docx]

Table S1.Database Search strategy

| MEDLINE via PubMed | ("patient reported experience"[Title/Abstract] OR "Patient Reported Experience Measure"[Title/Abstract] OR "PREM"[Title/Abstract]) AND ("Community Care"[Title/Abstract] OR "community care nurs*"[Title/Abstract] OR "Health care service"[Title/Abstract] OR "Primary Care"[Title/Abstract] OR "Primary Care Nursing"[Title/Abstract] OR "Domiciliary Care"[Title/Abstract] OR "territorial assistance"[Title/Abstract] OR "territorial healthcare"[Title/Abstract] OR "territorial care"[Title/Abstract] OR ("Community Health Nursing"[MeSH Terms] OR "Primary Health Care"[MeSH Terms] OR "Home Care Services"[MeSH Terms])) |
| --- | --- |
| CINAHL (EBSCO) | XB (“patient reported experience” OR (“patient-reported experience measures” or “prem”)) AND XB (((“community care” OR “community care nurs*” OR “health care services” OR “primary care” OR “primary care nursing” OR “domiciliary care” OR “territorial assistance” OR “territorial healthcare” OR “territorial care”) OR (MH "Community Health Nursing" OR MH "Primary Health Care" OR MH "Home Health Care"))) |
| PsycINFO (EBSCO) | XB (("patient reported experience" OR "patient-reported experience measures" OR "prem")) AND XB ((("community care" OR "community care settings" OR "community care nurs*" OR "community health services" OR "primary care" OR “primary care nursing” OR "domiciliary care" OR "home health care" OR "health care delivery" OR “territorial assistance” OR “territorial healthcare” OR “territorial care”)) OR (DE "Primary Health Care" OR DE "Health Care Delivery" OR DE "Nursing Homes" OR DE "Nursing Home Residents" OR DE "Residential Care" OR DE "Health Services" OR DE "Health Care Services" OR DE "Community Services" OR DE "Mental Health" OR DE "Long Term Care" OR DE "Home Care Personnel")) |
| SCOPUS | (TITLE-ABS-KEY ("patient reported experience" OR "Patient Reported Experience Measure" OR "PREM") AND TITLE-ABS-KEY (("Community Care" OR "community care nurs*" OR "Health care service" OR "Primary Care" OR "Primary Care Nursing" OR "Domiciliary Care" OR "territorial assistance" OR "territorial healthcare" OR "territorial care"))) |
| WEB OF SCIENCE | TS=("patient reported experience" OR "Patient Reported Experience Measure" OR "PREM") AND TS=(("Community Care" OR "community care nurs*" OR "Health care service" OR "Primary Care" OR "Primary Care Nursing" OR "Domiciliary Care" OR "territorial assistance" OR "territorial healthcare" OR "territorial care" OR "Community Health Nursing" OR "Primary Health Care" OR "Home Care Services")) |
