# Supplementary material for: Patient-reported experiences and patient-reported experience measures in primary care, home care, and community settings. A scoping review
Source: Front Rehabil Sci. 2026 Sep 24;7:1966101. doi: 10.3389/fresc.2026.1966101 (PMC13646105; doi:10.3389/fresc.2026.1966101)
Supplement: Supplementary file 2 [file Table2.docx]

**Table S2**. Characteristics of the included studies and PREM instruments.

| **First author (Year)** | **Country** | **Geographic macroarea** | **Study type** | **Study design** | **Care setting** | **Clinical condition** | **Population** | **Age group** | **PREM instrument** | **Instrument type** |
| --- | --- | --- | --- | --- | --- | --- | --- | --- | --- | --- |
| Harrison et al., 2024 | Australia; Canada; Estonia; France; Germany; Hong Kong; Ireland; Norway; Netherlands; United Kingdom; Spain; United States | Asia; Europe; North America; Oceania | Review study | Mixed-methods | Mixed settings | Intellectual disability |  | Mixed | AASPIRE Healthcare Experiences Survey; Person-Centred Coordinated Care Experience Questionnaire (P3CEQ); Ad hoc instrument | Validated PREMs and ad hoc instruments |
| Patel et al., 2025 | United Kingdom | Europe | Primary study | Quantitative | Mixed settings | Hearing disorders |  | Adults | UK Audiology Care Questionnaire (CQI-based) | Validated PREMs only |
| Soto‐Rubio et al., 2024 | Spain | Europe | Primary study | Quantitative | Mixed settings |  | General clinical population | Adults | Sinclair Compassion Questionnaire (SCQ) | Validated PREMs only |
| Sidey-Gibbons et al., 2019 | United Kingdom | Europe | Primary study | Quantitative | Community services | Mental health and substance use disorders |  | Adults | EQUIP Physical Health PREM (EQUIP-PH-PREM) | Validated PREMs only |
| Migchels et al., 2023 | Australia; Bulgaria; Germany; Norway; United States; South Africa | Africa; Europe; North America; Oceania | Review study | Mixed-methods | Mixed settings | Mental health and substance use disorders |  | Adults | Experiences of Care and Health Outcomes Survey (ECHO); South African Addiction Treatment Services Assessment (SAATSA); Community Oriented Programs Environment Scale (COPES); Primary Care Assessment Survey (PCAS); National Quality Register for Substance Abuse Treatment (NQR-SAT / KVARUS) | Validated PREMs only |
| Olding et al., 2018 | Canada | North America | Primary study | Qualitative | Mixed settings | Mental health and substance use disorders |  |  | Ad hoc instrument | Ad hoc instruments only |
| Rodríguez-Fuertes et al., 2023 | Spain | Europe | Primary study | Mixed-methods | Mixed settings | Postoperative conditions |  | Adults | Ad hoc instrument | Ad hoc instruments only |
| Browne et al., 2024 | Canada | North America | Primary study | Quantitative | Mixed settings |  | Vulnerable population | Adults | Equity-Oriented Health Care Scale – Ongoing (EOHCS-Ongoing); Equity-Oriented Health Care Scale – Episodic (EOHCS-Episodic) | Validated PREMs only |
| Williams et al., 2018 | United Kingdom | Europe | Primary study | Quantitative | Mixed settings | Neurological disorders |  | Pediatric | Ad hoc instrument | Ad hoc instruments only |
| van der Sterren et al., 2023 | Australia; Canada; Estonia; Germany; Ireland; Italy; Norway; Netherlands; Portugal; United Kingdom; Spain; United States; South Africa; Switzerland; Ukraine | Africa; Europe; North America; Oceania | Review study | Qualitative | Mixed settings | Mental health and substance use disorders |  | Adults | Patient-Reported Experience Measure for Addiction Treatment (PREMAT); Patient Experience Questionnaire for Interdisciplinary Treatment for Substance Dependence (PEQ-ITSD); Service Users’ Satisfaction and Outcomes Survey (SUSOS); Client Perception of Care (CPC); Addiction and Mental Health Patient Experience Survey; Alcohol and Other Drug Experience Questionnaire (AOD Experience Questionnaire); Experiences of Patient-Centred Care Questionnaire | Validated PREMs only |
| Maini et al., 2018 | United Kingdom | Europe | Primary study | Quantitative | Mixed settings | Neurological disorders |  | Pediatric | Epilepsy12 Patient/Carer and Child Experience Questionnaire (Epilepsy12 PREM) | Validated PREMs only |
| Loos et al., 2025 | Netherlands | Europe | Primary study | Quantitative | Primary care | Musculoskeletal disorders |  | Adults | PREM Ziekenhuiszorg (Dutch Hospital Care PREM) | Validated PREMs only |
| Kim et al., 2023 | South Korea | Asia | Primary study | Quantitative | Mixed settings |  | General clinical population | Adults | Korea Health Panel Survey (KHPS) | Validated PREMs only |
| Schick-Makaroff et al., 2021 | Australia; Belgium; Canada; Finland; Japan; Norway; New Zealand; Netherlands; United Kingdom; Spain; United States; Sweden | Asia; Europe; North America; Oceania | Review study | Qualitative | Mixed settings |  | Frail older adults | Older adults | CANHELP Lite – Patient Version; CANHELP Lite – Family Version; OARS Client Satisfaction Questionnaire; Ad hoc instrument | Validated PREMs and ad hoc instruments |
| Arnold et al., 2022 | Germany | Europe | Primary study | Quantitative | Primary care | Cardiovascular diseases |  | Adults | Nijmegen Continuity Questionnaire (NCQ); Ad hoc instrument | Validated PREMs and ad hoc instruments |
| Ljungholm et al., 2024 | Sweden | Europe | Primary study | Quantitative | Primary care | Unspecified chronic conditions |  | Adults | Patient Experienced Continuity of Care Questionnaire (PECQ) | Validated PREMs only |
| Humphries et al., 2021 | Canada | North America | Primary study | Quantitative | Primary care | Cardiovascular diseases |  | Adults | Ad hoc instrument | Ad hoc instruments only |
| Duhoux et al., 2022 | Canada | North America | Primary study | Quantitative | Primary care |  | Vulnerable population | Adults | Patient Experience Questionnaire in Primary Care (PEQ-PC) | Validated PREMs only |
| Lukewich et al., 2022 | Australia; New Zealand; United Kingdom; United States | Europe; North America; Oceania | Review study | Quantitative | Primary care |  | General clinical population | Adults | Patient Satisfaction Questionnaire (PSQ); Visit-Specific Satisfaction Questionnaire (VSQ-9); Primary Care Assessment Survey (PCAS); Primary Care Assessment Tool (PCAT); Client Satisfaction Questionnaire (CSQ-8); Patient Enablement Instrument (PEI); Nurse–Patient Interaction Scale (NPIS); Quality of Care from the Patient’s Perspective (QPP); General Practice Assessment Questionnaire (GPAQ) | Validated PREMs only |
| Wood et al., 2022 | United Kingdom | Europe | Primary study | Qualitative | Primary care | Musculoskeletal disorders |  | Adults | Friends and Family Test (FFT) | Validated PREMs only |
| Kilpatrick et al., 2021 | Canada | North America | Primary study | Quantitative | Primary care |  | General clinical population | Mixed | Patient-PTE Questionnaire (PTE) | Validated PREMs only |
| Sjetne & Iversen, 2017 | Norway | Europe | Primary study | Quantitative | Mixed settings | Maternal health |  | Adults | Pregnancy and Maternity Care Patients’ Experiences Questionnaire (PreMaPEQ) | Validated PREMs only |
| Wild HM et al., 2024 | United Kingdom | Europe | Review study | Qualitative | Mixed settings | Kidney disease |  | Mixed | Kidney Patient-Reported Experience Measure (Kidney PREM); Kidney Patient-Reported Experience Measure – Short Form (Kidney PREM-SF); Needling Patient-Reported Experience Measure (Needling PREM); Pediatric Kidney Patient-Reported Experience Measure (Pediatric Kidney PREM) | Validated PREMs only |
| Weinhandl ED & Forfang D, 2022 | United States | North America | Review study | Qualitative | Mixed settings | Kidney disease |  | Adults | In-Center Hemodialysis Consumer Assessment of Healthcare Providers and Systems (ICH CAHPS); Home Dialysis Care Experience Instrument (Home-DCE); Ad hoc instrument | Validated PREMs and ad hoc instruments |
| Burch et al., 2024 | Australia; Canada; Japan; India; Israel; Norway; Netherlands; United Kingdom; United States | Asia; Europe; North America; Oceania | Review study | Quantitative | Mixed settings |  | General clinical population | Adults | Nijmegen Continuity Questionnaire (NCQ); Continuity of Care Across Levels Questionnaire (CCAENA); Patient Continuity of Care Questionnaire (PCCQ); Alberta Continuity of Care Survey (ACS); Patient-Perceived Continuity of Care Scale (Chao); Primary Care Assessment Tool (PCAT); Care Continuity Index (CCI); Ad hoc instrument | Validated PREMs and ad hoc instruments |
| O’Loughlin M et al., 2017 | United States | North America | Review study | Mixed-methods | Mixed settings | Unspecified chronic conditions |  | Adults | Clinician & Group Consumer Assessment of Healthcare Providers and Systems (CG-CAHPS); Patient Assessment of Chronic Illness Care (PACIC); Primary Care Assessment Survey (PCAS); Ambulatory Care Experiences Survey – Short Form (ACES-SF); Components of Primary Care Index (CPCI); Survey of Healthcare Experiences of Patients (SHEP); Picker Patient Experience Questionnaire (PPEQ); Press Ganey Patient Experience Survey; Patient Activation Measure (PAM); Health Center Patient Survey; Diabetes Disease State Management Questionnaire – Modified (DDSM-QM); Consumer Choice Insurer Satisfaction Survey; HealthPartners Medical Group Patient Survey; Alberta Continuity of Care Survey (ACS) | Validated PREMs only |
| Teale EA & Young JB, 2015 | United Kingdom | Europe | Primary study | Quantitative | Mixed settings |  | Frail older adults | Older adults | Intermediate Care Patient-Reported Experience Measures – Bed-based (IC-PREMs); Intermediate Care Patient-Reported Experience Measures – Home-based (IC-PREMs) | Validated PREMs only |
| Luft HS et al., 2020 | United States | North America | Primary study | Quantitative | Primary care |  | General clinical population | Adults | Press Ganey Outpatient Medical Practice Survey | Validated PREMs only |
| Davis EL et al., 2020 | Australia; Canada; Denmark; France; Norway; United Kingdom; United States; Thailand | Asia; Europe; North America; Oceania | Review study | Qualitative | Mixed settings | Mental health and substance use disorders |  | Adults | Treatment Perceptions Questionnaire (TPQ); Texas Christian University Client Evaluation Form (TCU-CEF); Client Evaluation of Self and Treatment (CEST); Patient Involvement Scales (PIS); Helping Alliance Questionnaire (HAQ); Ad hoc instrument | Validated PREMs and ad hoc instruments |
| Giannopoulos E et al., 2024 | Canada | North America | Primary study | Quantitative | Mixed settings | Cancer |  | Adults | Ad hoc instrument | Ad hoc instruments only |
| Blood Z et al., 2021 | France; Germany; Italy; Netherlands; United Kingdom; Spain; United States | Europe; North America | Review study | Qualitative | Mixed settings | Cancer |  |  | EORTC Quality of Life Questionnaire – Information Module (EORTC QLQ-INFO26) | Validated PREMs only |
| Goldthorpe J et al., 2018 | United Kingdom | Europe | Primary study | Mixed-methods | Primary care | Postoperative conditions |  | Adults | Ad hoc instrument | Ad hoc instruments only |
| Lovén M et al., 2024 | Finland | Europe | Primary study | Quantitative | Community services | Dermatological disorders |  | Adults | Patient Enablement Instrument (PEI); Net Promoter Score (NPS); Ad hoc instrument | Validated PREMs and ad hoc instruments |
| Luckett T et al., 2022 | Australia | Oceania | Primary study | Qualitative | Mixed settings | Palliative care |  | Adults | Ad hoc instrument | Ad hoc instruments only |
| Howard AF et al., 2024 | Canada | North America | Primary study | Qualitative | Primary care |  | General clinical population | Adults | Ad hoc instrument | Ad hoc instruments only |
| Rivara MB et al., 2024 | United States | North America | Primary study | Quantitative | Home care | Kidney disease | Multiethnic groups | Adults | Home Dialysis Care Experience (Home-DCE); Quality of Home Dialysis Center Care and Operations Scale | Validated PREMs only |
| Aysola J et al., 2020 | United States | North America | Primary study | Quantitative | Primary care | Multiple chronic conditions |  | Adults | Press Ganey Outpatient Medical Practice Survey | Validated PREMs only |
| Jackson K & Newbury-Birch D, 2024 | Australia; Canada; Denmark; Finland; France; Germany; Norway; Netherlands; United States | Europe; North America; Oceania | Review study | Quantitative | Mixed settings | Neurological disorders |  | Adults | Parkinson's Disease Care Questionnaire (PCQ-PD); Patient Assessment of Chronic Illness Care (PACIC); Patient Care Questionnaire for Rehabilitation Services (PCQRS); Experience of Care and Health Outcomes (ECHO); Health Information National Trends Survey (HINTS); Quality of Care from the Patient's Perspective (QUOTE-EEE); Outpatient Experience Questionnaire (OPEQ); Patient Satisfaction Questionnaire Short Form (PSQ-18); Consultation and Relational Empathy Measure (CARE Measure); Patient Questionnaire for Rehabilitation Services (PQRS) | Validated PREMs only |
| Spitaels D et al., 2020 | Belgium | Europe | Primary study | Quantitative | Primary care | Musculoskeletal disorders |  | Adults | European Task Force on Patient Evaluations of General Practice Care (EUROPEP) | Validated PREMs only |
| Aoki T et al., 2022 | Japan | Asia | Primary study | Quantitative | Mixed settings | Multiple chronic conditions |  | Adults | Japanese Primary Care Assessment Tool – Short Form (JPCAT-SF) | Validated PREMs only |
| Gleeson H et al., 2016 | Canada; Netherlands; United Kingdom; United States | Europe; North America | Review study | Qualitative | Mixed settings |  | General clinical population | Mixed | Consumer Assessment of Healthcare Providers and Systems (CAHPS); NHS Patient Survey; Ad hoc instrument | Validated PREMs and ad hoc instruments |
| Tint SS et al., 2025 | Denmark; Ecuador; France; Norway; United Kingdom; Sweden | Europe; South America | Review study | Qualitative | Mixed settings | Metabolic disorders |  | Mixed | Patient Experience of Diabetes Service Survey (PEDS); Type 1 Diabetes Youth Patient-Reported Experience Measure (T1D Youth PREM); Swedish National Diabetes Register Patient Experience Survey (NDR PREM); Adolescent Patient Experience Questionnaire for Diabetes Care (APEQ-DC); Outpatient Clinic Patient-Reported Experience Measure (Outpatient Clinic PREM); EDP Diabetes Patient Experience Questionnaire (EDP Questionnaire); Inpatient Diabetes Patient-Reported Experience Measure (Inpatient Diabetes PREM); Diabetes Reported Experience Measure Survey (DREMS) | Validated PREMs only |
| Tacchini-Jacquier N et al., 2024 | Switzerland | Europe | Primary study | Quantitative | Mixed settings | Infectious diseases |  | Adults | Ad hoc instrument | Ad hoc instruments only |
| Zigman Suchsland ML et al., 2019 | United States | North America | Primary study | Qualitative | Primary care |  | General clinical population | Adults | Ad hoc instrument | Ad hoc instruments only |
| L’Esperance V et al., 2021 | United Kingdom | Europe | Primary study | Quantitative | Primary care |  | General clinical population | Adults | GP Patient Survey (GPPS) | Validated PREMs only |
| Yoshimura M & Sumi N, 2022 | Australia; Brazil; Canada; China; South Korea; Japan; Ireland; Israel; Italy; Norway; United Kingdom; Singapore; United States; Sweden; Switzerland; Taiwan; Türkiye | Asia; Europe; North America; Oceania; South America | Review study | Quantitative | Mixed settings |  |  | Adults | Care Transitions Measure (CTM); Patient Continuity of Care Questionnaire (PCCQ); Discharge Care Experiences Survey (DICARES); B-PREPARED Discharge Readiness Scale (B-PREPARED); Partners at Care Transitions Measure (PACT-M) | Validated PREMs only |
| Burgess R et al., 2021 | United Kingdom | Europe | Primary study | Quantitative | Mixed settings | Musculoskeletal disorders |  | NR* | Friends and Family Test (FFT); Valuing Patients as Individuals; CollaboRATE; Musculoskeletal Care Indicators (MSK Indicators) | Validated PREMs only |
| Ämmälä AJ & Taimela S, 2024 | Finland | Europe | Primary study | Quantitative | Primary care |  | General clinical population | Adults | Customer Satisfaction Score (CSAT); Patient Enablement Instrument (PEI) | Validated PREMs only |
| Davis S et al., 2022 | Canada | North America | Primary study | Qualitative | Primary care | Mental health and substance use disorders |  | Adults | Kootenay Boundary Patient Experience Survey (KB Patient Experience Survey) | Validated PREMs only |
| Hollingworth W et al., 2025 | United Kingdom | Europe | Primary study | Quantitative | Primary care |  | General clinical population | Adults | Patient-Reported Experiences and Outcomes of Safety in Primary Care (PREOS-PC) | Validated PREMs only |
| Noort BAC et al., 2023 | Netherlands | Europe | Primary study | Mixed-methods | Mixed settings | Respiratory diseases |  | Adults | Chronic Care Patient-Reported Experience Measure (PREM-CC) | Validated PREMs only |
| Ballart X & Rico G, 2023 | Spain | Europe | Primary study | Quantitative | Primary care |  | General clinical population | Mixed | CatSalut Primary Care Patient Experience Survey (PREM CatSalut) | Validated PREMs only |
| Rijken M et al., 2022 | Netherlands | Europe | Primary study | Quantitative | Primary care | Multiple chronic conditions |  | Adults | Person-Centred Coordinated Care Experience Questionnaire (P3CEQ) | Validated PREMs only |
| Rosenlund L et al., 2022 | United Kingdom; Sweden | Europe | Primary study | Mixed-methods | Primary care | Unspecified chronic conditions |  | Adults | Person-Centred Care Item Bank (PCC-IB) | Validated PREMs only |
| Montgomery-Taylor S et al., 2016 | United Kingdom | Europe | Primary study | Mixed-methods | Primary care |  | General clinical population | Pediatric | Ad hoc instrument | Ad hoc instruments only |
| Caneiras C et al., 2022 | Portugal | Europe | Primary study | Qualitative | Home care | Respiratory diseases |  | Adults | Ad hoc instrument | Ad hoc instruments only |
| Migchels C et al., 2024 | Belgium | Europe | Primary study | Quantitative | Community services | Mental health and substance use disorders |  | Adults | Patient-Reported Experience Measure in Addiction Treatment (PREMAT-NL) | Validated PREMs only |
| Haynes CJ et al., 2024 | Australia; Canada; Estonia; Indonesia; Iran; Kenya; Norway; New Zealand; Puerto Rico; United Kingdom; Spain; United States; Ukraine | Africa; Asia; Europe; North America; Oceania | Review study | Mixed-methods | Mixed settings | Mental health and substance use disorders |  | Adults | Client Satisfaction Questionnaire (CSQ-8); Verona Service Satisfaction Scale for Methadone Treatment (VSSS-MT); Treatment Perceptions Questionnaire (TPQ); Helping Alliance Questionnaire (HAQ); Primary Care Buprenorphine Satisfaction Scale (PCBSS); Ad hoc instrument | Validated PREMs and ad hoc instruments |
| Gasser F et al., 2023 | Sweden | Europe | Primary study | Quantitative | Community services in hospital care | Multiple chronic conditions |  | Adults | Person-Centred Care Instrument for Outpatient Care (PCCoc) | Validated PREMs only |
| Baart A et al., 2025 | Australia; Canada; Finland; Germany; Japan; Iran; Netherlands; United Kingdom; United States; Sweden; Switzerland; Thailand | Asia; Europe; North America; Oceania | Review study | Mixed-methods | Mixed settings | Maternal health |  | Adults | Continuity of Care Questionnaire (CCQ); Nijmegen Continuity Questionnaire (NCQ); Patient Continuity Index (PCI); Consultation and Relational Empathy Measure (CARE Measure); Pregnancy and Childbirth Questionnaire (PCQ); Ad hoc instrument | Validated PREMs and ad hoc instruments |
| Howard FD et al., 2024 | Australia; Belgium; Brazil; Canada; Netherlands; United Kingdom; Spain; United States; Sweden | Europe; North America; Oceania; South America | Review study | Qualitative | Mixed settings | Palliative care | Frail older adults | Older adults | Measure Yourself Concerns and Wellbeing (MYCaW); consideRATE Patient Experience Measure; Carer Support Needs Assessment Tool (CSNAT); Adult Social Care Outcomes Toolkit for Carers (ASCOT-Carer) | Validated PREMs only |
| Blake PG & Brown EA, 2020 | Canada; United Kingdom | Europe; North America | Primary study | Qualitative | Mixed settings | Kidney disease |  |  | Patient Activation Measure (PAM); Ad hoc instrument | Validated PREMs and ad hoc instruments |
| Lloyd H et al., 2019 | United Kingdom | Europe | Primary study | Quantitative | Primary care | Multiple chronic conditions |  | Adults | Person-Centred Coordinated Care Experience Questionnaire (P3CEQ) | Validated PREMs only |
| Lamprell K et al., 2023 | Australia; United Kingdom; New Zealand | Europe; Oceania | Primary study | Qualitative | Mixed settings | Cancer |  | Adults | Ad hoc instrument | PRE without a specified instrument |
| Narayanan A et al., 2022 | Australia | Oceania | Primary study | Quantitative | Primary care |  | General clinical population | Adults | Doctor’s Interpersonal Skills Questionnaire (DISQ) | Validated PREMs only |
| Deville-Stoetzel N et al., 2024 | Canada | North America | Primary study | Quantitative | Primary care |  | General clinical population | Adults | Ad hoc instrument | Ad hoc instruments only |
| Etingen B et al., 2016 | United States | North America | Primary study | Quantitative | Community services in hospital care |  | Veterans | Adults | Patient Activation Measure (PAM); Combined Outcome Measure for Risk Communication and Treatment Decision Making (COMRADE); Consultation and Relational Empathy Measure (CARE Measure); Patient Assessment of Chronic Illness Care (PACIC); Press Ganey Outpatient Medical Practice Survey | Validated PREMs only |
| Eton DT et al., 2020 | United States | North America | Primary study | Mixed-methods | Mixed settings | Multiple chronic conditions |  | Adults | Patient Assessment of Chronic Illness Care (PACIC) | Validated PREMs only |
| Zeh S et al., 2019 | Germany | Europe | Primary study | Quantitative | Mixed settings | Multiple chronic conditions |  | Adults | Ad hoc instrument | Ad hoc instruments only |
| Nuti S et al., 2017 | Italy | Europe | Primary study | Qualitative | Mixed settings |  | General clinical population |  | Ad hoc instrument | Ad hoc instruments only |
| Willi D et al., 2025 | Switzerland | Europe | Primary study | Quantitative | Mixed settings | Cancer |  |  | Cancer Patient Experience Questionnaire – Switzerland (CAPE-CH) | Validated PREMs only |
| Lyratzopoulos G et al., 2012 | United Kingdom | Europe | Primary study | Quantitative | Primary care |  | General clinical population | Adults | GP Patient Survey (GPPS) | Validated PREMs only |
| Brito Fernandes Ó et al., 2020 | Hungary | Europe | Primary study | Quantitative | Mixed settings |  | General clinical population | Adults | OECD Standardised Patient-Reported Experience Measures (PREMs) | Validated PREMs only |
| Piraux A et al., 2024 | France | Europe | Primary study | Qualitative | Mixed settings | Multiple chronic conditions |  | Adults | Patient-Centered Coordination by a Care Team (PCCCT) | Validated PREMs only |
| Haex R et al., 2020 | Netherlands | Europe | Primary study | Qualitative | Home care |  | General clinical population | Adults | Ad hoc instrument | Ad hoc instruments only |
| Eton DT et al., 2024 | United States | North America | Primary study | Mixed-methods | Outpatient care | Multiple chronic conditions | Multiethnic groups | Adults | Patient Experience with Treatment and Self-Management (PETS) | Validated PREMs only |
| Barr PJ et al., 2017 | United States | North America | Primary study | Quantitative | Primary care |  | General clinical population | Adults | CollaboRATE | Validated PREMs only |
| van Zwol-Janssens C et al., 2024 | Netherlands | Europe | Primary study | Quantitative | Community services in hospital care | Gynecological disorders |  | Adults | Validated questionnaire | PRE without a specified instrument |
| Haile ST et al., 2025 | Sweden | Europe | Primary study | Quantitative | Community services in hospital care | Cardiovascular diseases |  | Older adults | Quality from the Patient’s Perspective (QPP) | Validated PREMs only |
| McErlane F et al., 2018 | United Kingdom | Europe | Primary study | Quantitative | Community services in hospital care | Musculoskeletal disorders |  | Mixed | Ad hoc instrument | Ad hoc instruments only |
| Cunningham R et al., 2024 | New Zealand | Oceania | Primary study | Quantitative | Primary care |  | General clinical population | Mixed | Primary Care Patient Experience Survey (PCPES) | Validated PREMs only |
| Marsall M et al., 2024 | Germany | Europe | Primary study | Quantitative | Mixed settings |  | General clinical population | Adults | Care Transitions Measure (CTM-19) | Validated PREMs only |
| Strohschein FJ et al., 2024 | Canada | North America | Primary study | Quantitative | Outpatient care | Cancer |  | Adults | Ambulatory Oncology Patient Satisfaction Survey (AOPSS) | Validated PREMs only |
| Christalle E et al., 2025 | Germany | Europe | Primary study | Quantitative | Community services in hospital care |  | General clinical population | Adults | Experience of Patient Assessment Tool – Short Form (EPAT-16) | Validated PREMs only |
| Hoepner JK et al., 2024 | Canada; United States | North America | Primary study | Quantitative | Mixed settings | Neurological disorders |  | Adults | Consultation and Relational Empathy Measure – TBI version (mCARE-TBI) | Validated PREMs only |
| White TM et al., 2024 | Spain | Europe | Primary study | Mixed-methods | Mixed settings | Infectious diseases |  | NR* | Ad hoc instrument | Ad hoc instruments only |
| Ho L et al., 2013 | Canada; United States | North America | Primary study | Quantitative | Primary care |  | General clinical population | Adults | Consumer Assessment of Healthcare Providers and Systems (CAHPS) | Validated PREMs only |
| Bonney et al., 2022 | Australia | Oceania | Primary study | Quantitative | Primary care | Multiple chronic conditions |  | Adults | Primary Care Assessment Tool – Short Form (PCAT-S) | Validated PREMs only |
| Breton et al., 2024 | Canada | North America | Primary study | Quantitative | Primary care |  | General clinical population | Adults | Ad hoc instrument | Ad hoc instruments only |
| Wagner et al., 2024 | Switzerland | Europe | Primary study | Quantitative | Home care | Neurological disorders |  | Adults | Ad hoc instrument | Ad hoc instruments only |
| Chung et al., 2020 | United States | North America | Primary study | Quantitative | Primary care |  | General clinical population | Adults | Clinician & Group Consumer Assessment of Healthcare Providers and Systems (CG-CAHPS) | Validated PREMs only |
| Gibson et al., 2025 | United Kingdom | Europe | Primary study | Quantitative | Primary care |  | General clinical population |  | GP Patient Survey (GPPS) | Validated PREMs only |
| Norman et al., 2024 | Norway | Europe | Primary study | Quantitative | Primary care |  | General clinical population | Adults | Patient Experience Questionnaire for General Practice (PEQ-GP) | Validated PREMs only |
| Cowell et al., 2024 | United Kingdom | Europe | Primary study | Quantitative | Primary care | Postoperative conditions |  | Adults | Ad hoc instrument | Ad hoc instruments only |
| Medina-Ranilla et al., 2023 | Argentina; Brazil; Chile; Colombia; Guatemala; Mexico; Peru; Puerto Rico | Central America; North America; South America | Review study | Qualitative | Mixed settings |  | General clinical population |  | National Health Survey – Brazil (Pesquisa Nacional de Saúde, PNS); National Quality of Life Survey – Colombia (Encuesta Nacional de Calidad de Vida); National Health and Nutrition Survey – Mexico (ENSANUT); IMSS Patient Experience Survey; Ad hoc instrument | Validated PREMs and ad hoc instruments |
| Jelin et al., 2024 | Norway | Europe | Primary study | Mixed-methods | Primary care |  | General clinical population | Adults | Validated questionnaire | PRE without a specified instrument |
| Abelsen et al., 2023 | Norway | Europe | Primary study | Mixed-methods | Primary care | Multiple chronic conditions | Vulnerable population |  | Ad hoc instrument | Ad hoc instruments only |
| Vaitkeviciene et al., 2023 | Lithuania | Europe | Primary study | Quantitative | Mixed settings | Multiple chronic conditions |  | Adults | Ad hoc instrument | Ad hoc instruments only |
| Bryant et al., 2023 | Australia | Oceania | Primary study | Qualitative | Community services in hospital care | Musculoskeletal disorders |  | Adults | Ad hoc instrument | Ad hoc instruments only |
| Lin et al., 2022 | Australia | Oceania | Primary study | Quantitative | Mixed settings | Postoperative conditions | Vulnerable population | Mixed | Ad hoc instrument | Ad hoc instruments only |
| Stynes et al., 2021 | United Kingdom | Europe | Primary study | Quantitative | Primary care | Musculoskeletal disorders |  | Adults | Friends and Family Test (FFT); Patient Enablement Instrument (PEI); CollaboRATE; Valuing Patients as Individuals | Validated PREMs only |
| Nadeau et al., 2024 | Canada | North America | Primary study | Mixed-methods | Mixed settings | Respiratory diseases |  | Adults | APEX Questionnaire | Validated PREMs only |
| Alasqah, 2023 | Saudi Arabia | Asia | Primary study | Quantitative | Primary care |  | General clinical population | Adults | Patient-Reported Experiences and Outcomes of Safety in Primary Care (PREOS-PC) | Validated PREMs only |
| Fiol-deRoque et al., 2024 | Spain | Europe | Primary study | Quantitative | Primary care |  | General clinical population | Mixed | Patient-Reported Experiences and Outcomes of Safety in Primary Care – Compact (PREOS-PC-Compact) | Validated PREMs only |
| Thomas et al., 2024 | United Kingdom | Europe | Primary study | Qualitative | Primary care | Musculoskeletal disorders |  | Adults | Patient-Reported Experiences and Outcomes of Safety in Primary Care (PREOS-PC) | Validated PREMs only |
| Savla et al., 2025 | United Kingdom | Europe | Primary study | Quantitative | Primary care | Postoperative conditions |  | Adults | Ad hoc instrument | Ad hoc instruments only |
| Serrano-Ripoll et al., 2022 | Spain | Europe | Primary study | Mixed-methods | Primary care |  | General clinical population |  | Patient-Reported Experiences and Outcomes of Safety in Primary Care (PREOS-PC); Medical Office Survey on Patient Safety Culture (MOSPSC) | Validated PREMs only |
| Huhtanen et al., 2025 | Australia | Oceania | Primary study | Quantitative | Community services in hospital care | Multiple chronic conditions |  | Pediatric | Ad hoc instrument | Ad hoc instruments only |
| Price et al., 2022 | United Kingdom | Europe | Primary study | Qualitative | Community services in hospital care |  | General clinical population | Mixed | Ad hoc instrument | Ad hoc instruments only |
| O’Keeffe et al., 2021 | United Kingdom | Europe | Primary study | Quantitative | Outpatient care | Neurological disorders |  | Adults | Experiences of Health and Social Care Questionnaire | Validated PREMs only |
| Hopstaken et al., 2021 | Australia; Belgium; Canada; Germany; Norway; Netherlands; United States | Europe; North America; Oceania | Review study | Mixed-methods | Mixed settings | Cancer |  | Adults | Ad hoc instrument | Ad hoc instruments only |
| d’Agincourt-Canning et al., 2024 | Canada | North America | Primary study | Qualitative | Mixed settings |  | Indigenous population |  | Hua Oranga; Native Wellness Assessment; Cultural Safety Survey; Cultural Connectedness Scale; Equity-Oriented Health Care Scale (EOHCS); Awareness of Connectedness Scale; Growth and Empowerment Measure (GEM); Multicultural Mastery Scale; Indigenous Peoples' Survey | Validated PREMs only |
| Tou et al., 2022 | United States | North America | Primary study | Qualitative | Primary care |  | Low socioeconomic status | Adults | Ad hoc instrument | Ad hoc instruments only |
| Siskou et al., 2025 | Greece | Europe | Primary study | Quantitative | Mixed settings |  | General clinical population | Adults | OECD Patient-Reported Indicator Surveys (PaRIS Survey) | Validated PREMs only |
| Nasser Albarqi, 2024 | Canada; China; Iran; Singapore; United States; Taiwan | Asia; North America | Review study | Qualitative | Mixed settings | Multiple chronic conditions |  | Older adults | Usual Provider of Care Index (UPC); Continuity of Care Index (COCI); Ad hoc instrument | Validated PREMs and ad hoc instruments |
| Maroni et al., 2022 | United Kingdom | Europe | Primary study | Mixed-methods | Primary care | Gastrointestinal disorders |  | Adults | Inventory to Assess Patient Satisfaction (IAPS) | Validated PREMs only |
| Ryan et al., 2025 | United Kingdom | Europe | Primary study | Mixed-methods | Community services in hospital care | Ophthalmologic disorders |  | Adults | Eye-Care Patient-Reported Experience Measure (Eye-Care PREM); Ad hoc instrument | Validated PREMs and ad hoc instruments |
| Fiol-deRoque et al., 2025 | Spain | Europe | Primary study | Mixed-methods | Primary care |  | General clinical population | Mixed | Patient-Reported Experiences and Outcomes of Safety in Primary Care – Compact (PREOS-PC-Compact) | Validated PREMs only |
| van der Sar et al., 2021 | Austria; Belgium; Bulgaria; France; Germany; Ireland; Italy; Norway; Netherlands; Poland; United Kingdom; Romania; Spain | Europe | Primary study | Quantitative | Mixed settings | Respiratory diseases |  | Adults | Ad hoc instrument | Ad hoc instruments only |
| Kutyla et al., 2023 | Australia | Oceania | Primary study | Quantitative | Outpatient care | Postoperative conditions |  | Adults | Comprehensive Endoscopy Satisfaction Tool (CEST) | Validated PREMs only |
| Valderas et al., 2024 | Ecuador; Finland; France; Germany; Netherlands; United Kingdom; Singapore; Spain | Latin America; Asia; Europe | Primary study | Qualitative | Primary care | Multiple chronic conditions |  | Adults | OECD Patient-Reported Indicator Surveys (PaRIS Survey) | Validated PREMs only |
| Barello et al., 2021 | Italy | Europe | Primary study | Mixed-methods | Mixed settings | Gastrointestinal disorders |  | Adults | WE-CARE IBD Score | Validated PREMs only |
| Torresdey et al., 2024 | United States | North America | Primary study | Quantitative | Community services |  | Population with language barriers | Adults | Clinician & Group Consumer Assessment of Healthcare Providers and Systems (CG-CAHPS) | Validated PREMs only |
| Coelho et al., 2022 | Portugal | Europe | Primary study | Quantitative | Mixed settings | Mental health and substance use disorders |  | Mixed | Ad hoc instrument | Ad hoc instruments only |
| Lin et al., 2022 | Canada | North America | Primary study | Qualitative | Community services in hospital care | Cardiovascular diseases |  | NR* | Ad hoc instrument | Ad hoc instruments only |
| Burgess et al., 2023 | United Kingdom | Europe | Review study | Quantitative | Mixed settings | Musculoskeletal disorders |  | Adults | National Musculoskeletal Patient-Reported Experience Measure (MSK PREM) | Validated PREMs only |
| Graham et al., 2021 | Australia; Canada; United States | North America; Oceania | Review study | Qualitative | Mixed settings | Multiple chronic conditions |  | Mixed | Ad hoc instrument | Ad hoc instruments only |
| Ruiz et al., 2025 | United States | North America | Primary study | Mixed-methods | Community services |  | Population with language barriers | Adults | Clinician & Group Consumer Assessment of Healthcare Providers and Systems (CG-CAHPS) | Validated PREMs only |
| Bajgain et al., 2020 | Canada | North America | Review study | Qualitative | Mixed settings |  | Multiethnic groups | Adults | Ad hoc instrument | Ad hoc instruments only |
| de Camargo Silva et al., 2024 | Brazil | Latin America | Primary study | Quantitative | Primary care |  | General clinical population | Adults | Patient-Reported Experiences and Outcomes of Safety in Primary Care – Compact (PREOS-PC-Compact Brazil) | Validated PREMs only |
| Fu et al., 2023 | United States | North America | Primary study | Mixed-methods | Primary care | Mental health and substance use disorders |  | Adults | Ad hoc instrument | Ad hoc instruments only |
| Paddison et al., 2018 | United Kingdom | Europe | Primary study | Quantitative | Primary care |  | General clinical population | Mixed | GP Patient Survey (GPPS) | Validated PREMs only |
| Grønhaug et al., 2014 | Norway | Europe | Primary study | Quantitative | Primary care | Musculoskeletal disorders |  | Adults | Osteoarthritis Quality Indicator Questionnaire (OA-QI) | Validated PREMs only |
| Valderas et al., 2019 | Australia; Canada; Ireland; New Zealand; Netherlands; United Kingdom; United States | Europe; North America; Oceania | Review study | Mixed-methods | Mixed settings | Multiple chronic conditions |  | NR* | Validated questionnaire | PRE without a specified instrument |
| Campbell et al., 2020 | United Kingdom | Europe | Primary study | Quantitative | Primary care |  | General clinical population | Adults | Patient-Reported Experiences and Outcomes of Safety in Primary Care (PREOS-PC) | Validated PREMs only |
| Pourmarzi et al., 2019 | Australia; Canada; France; Netherlands; Pakistan; United Kingdom; United States; Switzerland | Asia; Europe; North America; Oceania | Review study | Mixed-methods | Mixed settings | Infectious diseases |  | Adults | Ad hoc instrument | Ad hoc instruments only |
| Frahsa et al., 2020 | Switzerland | Europe | Primary study | Qualitative | Mixed settings |  | Multiethnic groups | Adults | Ad hoc instrument | Ad hoc instruments only |
| Doubova et al., 2020 | Mexico | Latin America | Primary study | Quantitative | Community services in hospital care | Cancer |  | Adults | Patient-Centered Quality of Cancer Care Questionnaire (PCQCCQ) | Validated PREMs only |
| Glenngård & Anell, 2018 | Sweden | Europe | Primary study | Quantitative | Primary care |  | General clinical population | Adults | National Patient Experience Survey (NPE) | Validated PREMs only |
| Cohidon et al., 2018 | Switzerland | Europe | Primary study | Quantitative | Primary care |  | General clinical population | Adults | Quality and Costs of Primary Care Questionnaire (QUALICOPC) | Validated PREMs only |
| Schäfer et al., 2019 | Australia; Austria; Belgium; Bulgaria; Canada; Cyprus; Denmark; Estonia; Finland; Germany; Greece; Ireland; Iceland; Italy; Latvia; Lithuania; Luxembourg; North Macedonia; Malta; Norway; New Zealand; Netherlands; Poland; Portugal; United Kingdom; Czech Republic; Romania; Slovakia; Slovenia; Spain; Sweden; Switzerland; Türkiye; Hungary | Europe; North America; Oceania | Review study | Quantitative | Primary care |  | General clinical population | NR* | Quality and Costs of Primary Care Questionnaire (QUALICOPC) | Validated PREMs only |
| Graham et al., 2007 | United Kingdom | Europe | Primary study | Mixed-methods | Primary care | Mental health and substance use disorders |  | Adults | Ad hoc instrument | Ad hoc instruments only |
| Hasnain et al., 2013 | United States | North America | Primary study | Quantitative | Primary care |  | General clinical population | Adults | Clinician & Group Consumer Assessment of Healthcare Providers and Systems (CG-CAHPS) | Validated PREMs only |
| Bradley et al., 2020 | United Kingdom | Europe | Primary study | Quantitative | Mixed settings | Postoperative conditions |  | Mixed | Orthodontic Patient Treatment Impact Questionnaire (OPTIQ) | Validated PREMs only |
| Philpot et al., 2019 | United States | North America | Primary study | Quantitative | Primary care | Internal medicine conditions |  | Adults | Press Ganey Medical Practice Survey | Validated PREMs only |
| Krist et al., 2008 | United States | North America | Primary study | Quantitative | Primary care |  | General clinical population | Adults | Ad hoc instrument | Ad hoc instruments only |
| Laws et al., 2019 | Canada | North America | Primary study | Quantitative | Mixed settings | Cancer |  | Adults | Ad hoc instrument | Ad hoc instruments only |
| Ahern et al., 2019 | Australia | Oceania | Primary study | Mixed-methods | Mixed settings | Musculoskeletal disorders |  | Adults | Ad hoc instrument | Ad hoc instruments only |
| Solheim et al., 2017 | Norway | Europe | Primary study | Quantitative | Community services in hospital care | Neurological disorders |  | Adults | PasOpp Patient Experience Questionnaire | Validated PREMs only |
| Ricci-Cabello et al., 2017 | United Kingdom | Europe | Primary study | Qualitative | Primary care | Internal medicine conditions |  | Adults | Patient-Reported Experiences and Outcomes of Safety in Primary Care (PREOS-PC) | Validated PREMs only |
| Zulman et al., 2019 | United States | North America | Primary study | Quantitative | Primary care |  | Veterans | Adults | Survey of Healthcare Experiences of Patients (SHEP); Patient Assessment of Chronic Illness Care (PACIC); Health Care Hassles Scale | Validated PREMs only |
| McDonald et al., 2018 | United States | North America | Primary study | Quantitative | Primary care | Multiple chronic conditions |  | Adults | Patient Assessment of Chronic Illness Care – Short Form (PACIC-11); CollaboRATE; Patient Activation Measure (PAM) | Validated PREMs only |
| Baker et al., 2015 | Denmark; Finland; France; Germany; Italy; Norway; United Kingdom; Spain; Sweden | Europe | Primary study | Mixed-methods | Mixed settings | Kidney disease |  | Adults | Ad hoc instrument | Ad hoc instruments only |
| Wallace et al., 2010 | United States | North America | Primary study | Quantitative | Outpatient care | Metabolic disorders |  | Adults | Patient Assessment of Chronic Illness Care (PACIC) | Validated PREMs only |
| Aysola et al., 2018 | United States | North America | Primary study | Quantitative | Outpatient care | Internal medicine conditions |  | Adults | Press Ganey Outpatient Medical Practice Survey | Validated PREMs only |
| Wong et al., 2019 | United States | North America | Primary study | Quantitative | Outpatient care |  | Veterans | Older adults | Survey of Healthcare Experiences of Patients (SHEP) | Validated PREMs only |
| Ricci-Cabello, Marsden, et al., 2017 | United Kingdom | Europe | Primary study | Quantitative | Primary care |  | General clinical population | Adults | Patient-Reported Experiences and Outcomes of Safety in Primary Care (PREOS-PC) | Validated PREMs only |
| Altin & Stock, 2015 | Germany | Europe | Primary study | Quantitative | Primary care |  | General clinical population | Adults | Commonwealth Fund International Health Policy Survey (IHP Survey) | Validated PREMs only |
| Ricci-Cabello, Reeves, et al., 2017 | United Kingdom | Europe | Primary study | Quantitative | Primary care |  | General clinical population | Adults | Patient-Reported Experiences and Outcomes of Safety in Primary Care (PREOS-PC) | Validated PREMs only |
| Crocker et al., 2017 | United States | North America | Primary study | Quantitative | Outpatient care |  | General clinical population | Adults | Consumer Assessment of Healthcare Providers and Systems (CAHPS); Ambulatory Care Experiences Survey (ACES); Consultation and Relational Empathy Measure (CARE Measure) | Validated PREMs only |
| Walker et al., 2017 | United Kingdom | Europe | Primary study | Qualitative | Mixed settings | Respiratory diseases |  | Adults | Ad hoc instrument | Ad hoc instruments only |
| Jackson et al., 2019 | Australia; Canada; Denmark; Ireland; Norway; New Zealand; Netherlands; United Kingdom; United States; South Africa; Sweden | Africa; Europe; North America; Oceania | Review study | Qualitative | Mixed settings | Neurological disorders |  | Adults | Ad hoc instrument | Ad hoc instruments only |
| Ricci-Cabello et al., 2016 | United Kingdom | Europe | Primary study | Quantitative | Primary care |  | General clinical population | Adults | Patient-Reported Experiences and Outcomes of Safety in Primary Care (PREOS-PC) | Validated PREMs only |
| Ricci-Cabello et al., 2021 | United Kingdom | Europe | Primary study | Mixed-methods | Primary care |  | General clinical population | Adults | Patient-Reported Experiences and Outcomes of Safety in Primary Care (PREOS-PC) | Validated PREMs only |
| Serrano-Ripoll, Llobera, et al., 2022 | Spain | Europe | Primary study | Mixed-methods | Primary care |  | General clinical population | Mixed | Patient-Reported Experiences and Outcomes of Safety in Primary Care – Compact (PREOS-PC-Compact) | Validated PREMs only |
| Mounce et al., 2022 | United Kingdom | Europe | Primary study | Quantitative | Primary care |  | General clinical population | Adults | Patient-Reported Experiences and Outcomes of Safety in Primary Care (PREOS-PC); Patient-Reported Experiences and Outcomes of Safety in Primary Care – Compact (PREOS-PC-Compact); Patient-Reported Experiences and Outcomes of Safety in Primary Care – Screen (PREOS-PC-Screen) | Validated PREMs only |
| Booker et al., 2025 | United Kingdom | Europe | Primary study | Mixed-methods | Primary care |  | General clinical population | NR* | Patient-Reported Experiences and Outcomes of Safety in Primary Care (PREOS-PC) | Validated PREMs only |
| Wang et al., 2025 | China | Asia | Primary study | Quantitative | Primary care |  | General clinical population | Adults | Primary Care Patient-Centered Measure (PCPCM) | Validated PREMs only |

**NR = Data not reported.*
