# Supplementary material for: Patient-reported experiences and patient-reported experience measures in primary care, home care, and community settings. A scoping review
Source: Front Rehabil Sci. 2026 Sep 24;7:1966101. doi: 10.3389/fresc.2026.1966101 (PMC13646105; doi:10.3389/fresc.2026.1966101)
Supplement: Supplementary file 3 [file Table3.docx]

**Table S3. Included PREMs instruments, pathology and target population**

| **Author/Year** | **PREMs Instrument** | **Main domains** | **Pathology** | **Population** | **Age group** |
| --- | --- | --- | --- | --- | --- |
| Harrison et al., 2024 | AASPIRE Survey; P3CEQ | Barriers to access; Unwelcoming care environments; Communication difficulties; Need for personalised support; Challenges during the transition from paediatric to adult care | Intellectual disability | - | Mixed |
| Patel et al., 2025 | UK Audiology CQI | Accommodation and facilities; Staff conduct; Staff competence; Appointment scheduling; Waiting times for appointments; Patient involvement; Treatment effectiveness | Hearing disorders | - | Adults |
| Soto‐Rubio et al., 2024 | SCQ | Feeling cared for; Feeling genuinely valued; Respectful and sensitive communication; Attentiveness to individual needs; Emotional comfort and reassurance; Support throughout care; Compassionate and appropriate care; Kind and respectful communication; Being treated as a person rather than just a patient; Caring and welcoming attitude; Understanding of patients' needs; Positive therapeutic relationship; Understanding of patients' perspectives; Warm and reassuring presence; Authentic and honest relationship | - | General clinical population | Adults |
| Sidey-Gibbons et al., 2019 | EQUIP-PH-PREM | Perceived involvement in the physical care plan; Communication with the care team; Equal attention to physical and mental health (parity of esteem); Access to information and self-management support | Mental health and substance use disorders | - | Adults |
| Migchels et al., 2023 | ECHO; SAATSA; COPES; PCAS; NQR-SAT/KVARUS | Satisfaction with treatment; Involvement in the treatment process; Therapeutic relationship; Respect; Perceived support; Trust in healthcare professionals; Perceived quality of care; Accessibility of services | Mental health and substance use disorders | - | Adults |
| Browne et al., 2024 | EOHCS-Ongoing | Welcoming and comfortable care environment; Accessibility and reduction of barriers; Individualised care tailored to patients' context, history, and experiences; Emotional safety and trust; Non-discriminatory care | - | Vulnerable population | Adults |
| Browne et al., 2024 | EOHCS-Episodic | Feeling welcomed; Feeling comfortable during care; Courteous and respectful treatment; Absence of discrimination; Feeling listened to regarding what matters most; Understanding of patients' individual circumstances; Appropriateness of advice provided; Assessment of barriers to accessing care; Support in accessing needed services | - | Vulnerable population | Adults |
| van der Sterren et al., 2023 | PREMAT; PEQ-ITSD; SUSOS; CPC; Addiction and Mental Health Patient Experience Survey; AOD Experience Questionnaire; Experiences of Patient-Centred Care Q. | Accessibility of services; Physical care environment; Relationships with healthcare professionals; Patient participation and involvement in decision-making; Non-discriminatory and stigma-free care; Cultural safety; Perceived support; Overall treatment experience and satisfaction; Continuity and coordination of care; Individualised care | Mental health and substance use disorders | - | Adults |
| Maini et al., 2018 | Epilepsy12 PREM | Provision of sufficient information about epilepsy; Clear and understandable communication; Feeling listened to; Staff taking time to understand patients; Involvement in decision-making; Respect for privacy; Confidence in staff competence; Opportunity to ask questions; Ease of contacting the epilepsy care team; Accessibility of clinic appointments; Appropriate frequency of follow-up; Timely communication about appointment delays; Age-appropriate waiting environment; Adequate consultation time; Coordination with other healthcare and educational services; Friendly and courteous staff interactions across care settings | Neurological disorders | - | Pediatric |
| Loos et al., 2025 | Dutch Hospital Care PREM | Shared decision-making; Discussion of the benefits and risks of treatment or surgery; Clear and understandable explanations; Perceived physician competence; Adequate consultation time; Opportunity to ask questions; Feeling taken seriously by the physician; Collaboration among healthcare team members | Musculoskeletal disorders | - | Adults |
| Kim et al., 2023 | KHPS | Accuracy of information provided; Physician–patient communication; Courtesy and respect from healthcare professionals; Overall experience with care received; Experience with treatment; Overall patient satisfaction | - | General clinical population | Adults |
| Schick-Makaroff et al., 2021 | CANHELP Lite (Patient); CANHELP Lite (Family); OARS CSQ | Access to care; Communication with healthcare professionals; Care coordination and continuity of care; Family support and caregiver involvement; Perceived quality of care transitions; Overall satisfaction with services received; Satisfaction with end-of-life care; Support throughout the care journey; Care environment and comfort; Interpersonal relationships with healthcare professionals and family members; Overall perceived quality of care; Communication and shared decision-making | - | Frail older adults | Adults |
| Arnold et al., 2022 | NCQ | Relational continuity with healthcare professionals; Personal knowledge of the patient by the physician; Communication and collaboration among healthcare professionals; Care coordination | Cardiovascular diseases | - | Adults |
| Ljungholm et al., 2024 | PECQ | Clear and understandable health information; Consistency of information provided; Information about follow-up care; Understanding of test results; Information continuity among healthcare professionals; Involvement of family members in communication; Healthcare professionals' familiarity with patients' medical history; Feeling safe with the care received; Relational continuity with healthcare professionals; Feeling taken seriously by healthcare professionals; Knowing who is responsible for care; Holistic assessment of patients' health needs; Regular follow-up of health conditions and treatments; Support with care coordination; Timely access to care and treatment; Shared care goals; Involvement in care planning; Support for self-management; Knowing whom to contact with questions; Person-centred care; Verification of patients' understanding; Access to understandable health information | Unspecified chronic conditions | - | Adults |
| Duhoux et al., 2022 | PEQ-PC | Accessibility of care; Continuity of care; Comprehensiveness of care; Responsiveness and perceived effectiveness of care; Perceived health outcomes of care | - | Vulnerable population | Adults |
| Lukewich et al., 2022 | PSQ; VSQ-9; PCAS; PCAT; CSQ-8; PEI; NPIS; QPP; GPAQ | Accessibility of care and waiting times; Communication and relationship with the registered nurse; Care coordination and continuity of care; Involvement in decision-making and patient empowerment; Perceived competence of and trust in the registered nurse | - | General clinical population | Adults |
| Wood et al., 2022 | FFT | Communication and clarity of information; Clinician competence, empathy, active listening, and respect; Perceived value of treatment and diagnosis; Efficiency and timely access to care; Favourable comparison with general practitioner (GP) care | Musculoskeletal disorders | - | Adults |
| Kilpatrick et al., 2021 | PTE | Clarity of team members' roles; Quality of team functioning; Perceived care outcomes; Communication; Team problem-solving; Focus on patients' and families' needs | - | General clinical population | Mixed |
| Sjetne & Iversen, 2017 | PreMaPEQ | Courtesy, respect, trust, cultural sensitivity, and involvement in decision-making; Completeness and clarity of information on maternal health, childbirth, breastfeeding, and follow-up care; Continuity of care across healthcare settings; Staff availability, time dedicated to patients, and organisational efficiency; Recognition of the partner's role during pregnancy and childbirth; Feeling of safety, psychological support, and comfort of the care environment; Accessibility and quality of municipal follow-up care, including breastfeeding and newborn care support | Maternal health | - | Adults |
| Wild HM et al., 2024 | Kidney PREM; Kidney PREM-SF; Needling PREM; Pediatric Kidney PREM | Access to services; Perceived support; Communication with the care team; Information provided; Dietary and fluid management; Medication management; Care planning and organisation; Privacy and dignity; Transportation; Care environment; Involvement in decision-making; Overall care experience | Kidney disease | - | Adults |
| Weinhandl ED & Forfang D, 2022 | ICH CAHPS; Home-DCE | Communication; Care coordination; Patient education; Comfort; Technical support; Caregiver burden | Kidney disease | - | Adults |
| Burch P et al., 2024 | NCQ; CCAENA; PCCQ; ACS; Chao; PCAT; CCI | Relational continuity with healthcare professionals; Information continuity across healthcare professionals and services; Care coordination and continuity of care; Patient empowerment; Perceived continuity of the care pathway; Treatment adherence; Reduced fragmentation of care | - | General clinical population | Adults |
| O’Loughlin M et al., 2017 | CG-CAHPS; PACIC; PCAS; ACES-SF; CPCI; SHEP; PPEQ; Press Ganey Patient Exp. Survey; PAM; Health Center Patient Survey; DDSM-QM; Consumer Choice Insurer Satisfaction Survey; HealthPartners Medical Group Patient Survey; ACS | Communication with physicians and the care team; Access to services; Care coordination; Self-management support; Involvement in decision-making; Relational continuity with healthcare professionals; Perceived quality of care | Unspecified chronic conditions | - | Adults |
| Teale EA & Young JB, 2015 | IC-PREMs (Bed-based) | Healthcare professionals' knowledge of patients' clinical history; Involvement in goal setting; Having questions answered; Trust in healthcare professionals; Involvement in discharge planning; Consideration of patients' home circumstances; Information provided to family members; Respect for patients' dignity | - | Frail older adults | Older adults |
| Teale EA & Young JB, 2015 | IC-PREMs (Home-based) | Healthcare professionals' knowledge of patients' clinical history; Awareness of care goals; Involvement in goal setting; Knowing how to contact healthcare professionals; Having questions answered; Trust in healthcare professionals; Involvement in discharge planning; Advance notice of discharge; Information provided to family members; Discussion of equipment needs after discharge; Discussion of post-discharge support services; Respect for patients' dignity and respect | - | Frail older adults | Older adults |
| Luft HS et al., 2020 | Press Ganey Outpatient Survey | Courtesy; Time dedicated to patients; Clear explanations and attention to patients' concerns | - | General clinical population | Adults |
| Davis EL et al., 2020 | TPQ; TCU-CEF; Client Evaluation of Self and Treatment (CEST); PIS; HAQ | Respect for patients' values, preferences, and needs; Care coordination and integration; Information and education; Physical comfort; Emotional support; Involvement of family and friends; Continuity of care and care transitions; Access to care | Mental health and substance use disorders | - | Adults |
| Blood Z et al., 2021 | EORTC QLQ-INFO26 | Amount of information received; Quality of information provided; Communication methods; Overall satisfaction with information and involvement in decision-making; Practical support; Management of treatment side effects; Availability of healthcare professionals to answer questions | Cancer | - | Adults |
| Lovén M et al., 2024 | PEI; NPS | Improved ability to cope with the illness; Greater overall satisfaction; Perceived timeliness of diagnosis and treatment; Positive experiences with communication and involvement in care | Dermatological disorders | - | Adults |
| Rivara MB et al., 2024 | Home-DCE; Home Dialysis Center Care and Operations Scale | Respect, active listening, and support from healthcare professionals; Clear communication and availability of healthcare professionals; Support with problem-solving and collaboration between healthcare professionals and physicians; Involvement in treatment decision-making and trust in the care team; Overall satisfaction with the quality and organisation of the care centre | Kidney disease | Multiethnic groups | Adults |
| Aysola J et al., 2020 | Press Ganey Outpatient Survey | Access to care; Visit management; Support staff; Care provided by the healthcare provider; Safety and privacy; Clarity of explanations; Involvement in decision-making; Time dedicated by the healthcare provider | Multiple chronic conditions | - | Adults |
| Jackson K & Newbury-Birch D, 2024 | PCQ-PD; PACIC; PCQRS; ECHO; HINTS; QUOTE-EEE; OPEQ; PSQ-18; CARE Measure; PQRS | Accessibility of care; Care coordination and continuity of care; Caregiver involvement; Information provided; Involvement in decision-making; Empathy and emotional support | Neurological disorders | - | Adults |
| Spitaels D et al., 2020 | EUROPEP | Patient–physician relationship and communication; Listening, interest, clear explanations, and patient involvement; Clinical competence and medical care; Pain and symptom management; Information about the disease, treatment, and prevention; Encouragement and support; Care coordination and continuity of care; Follow-up care; Accessibility of care; Waiting times; Physician availability and accessibility | Musculoskeletal disorders | - | Adults |
| Aoki T et al., 2022 | JPCAT-SF | First-contact access to care; Relational continuity and trust in the primary care physician; Care coordination; Comprehensiveness of available services; Comprehensiveness of services provided; Community orientation | Multiple chronic conditions | - | Adults |
| Gleeson H et al., 2016 | CAHPS; NHS Patient Survey | Overall satisfaction and patient–physician communication; Accessibility of care and waiting times; Care coordination and continuity of care; Information and educational materials provided; Emotional experiences and therapeutic relationships | - | General clinical population | Adults |
| Tint SS et al., 2025 | PEDS; T1D Youth PREM; NDR PREM; APEQ-DC; Outpatient Clinic PREM; EDP Questionnaire; Inpatient Diabetes PREM (Inpatient Diabetes PREM); DREMS | Care planning and goal setting; Patient education and self-management support; Professional competence, respect, empathetic communication, confidentiality, and honesty; Quality of care; Hospital care and care transitions | Metabolic disorders | - | Adults |
| L’Esperance V et al., 2021 | GPPS | Access to care; Relational continuity with the general practitioner; General practitioner professionalism; Nurse professionalism; Reception staff professionalism; Overall patient experience | - | General clinical population | Adults |
| Yoshimura M & Sumi N, 2022 | CTM; PCCQ; DICARES; B-PREPARED; PACT-M | Self-management after discharge; Information provided to patients; Involvement in care planning; Health status after discharge; Management of patients' concerns and questions; Care coordination across healthcare settings; Emotional and social support; Medication management; Readiness for discharge; Feeling safe and reassured at home; Family involvement | - | - | Adults |
| Burgess R et al., 2021 | FFT; Valuing Patients as Individuals; CollaboRATE; MSK Indicators | Respectful and compassionate care; Feeling listened to and valued as a person; Trust in healthcare professionals' clinical competence; Shared decision-making; Clear and comprehensive information; Perceived improvement in health status; Understanding of one's health condition; Self-efficacy and self-management; Overall experience with the service; Timeliness and convenience of care | Musculoskeletal disorders | - | NR |
| Ämmälä AJ & Taimela S, 2024 | CSAT; PEI | Feeling listened to by the physician; Receiving answers to patients' questions and concerns; Clear treatment and follow-up instructions | - | General clinical population | Adults |
| Davis S et al., 2022 | KB Patient Experience Survey | Ease of use and perceived value of the patient portal; Feeling listened to and understood; Frustration with healthcare professionals not reviewing patient-reported data; Value of personalised educational resources; Difficulties using the patient portal during periods of worsening health | Mental health and substance use disorders | - | Adults |
| Hollingworth W et al., 2025 | PREOS-PC | Well-being and trust in the healthcare system; Understanding of health status and care plan; Trust in the treatment plan; Perceived safety and safety promotion; Communication and accessibility of care | - | General clinical population | Adults |
| Noort BAC et al., 2023 | PREM-CC | Openness and clarity of explanations; Advice and support received; Involvement in decision-making; Perceived competence of healthcare professionals; Perceived effectiveness of health coaching; Care coordination among healthcare professionals; Overall experience with the service | Respiratory diseases | - | Adults |
| Ballart X & Rico G, 2023 | PREM CatSalut | Accessibility and timeliness of appointments; Communication and relationship with the physician; Care coordination among healthcare professionals; Courtesy and respectful treatment by healthcare professionals; Trust in healthcare professionals and perceived support; Care environment | - | General clinical population | Mixed |
| Rijken M et al., 2022 | P3CEQ | Involvement in decision-making; Holistic approach to the person; Care coordination and a designated care coordinator; Individualised care planning; Self-management support and information; Trust in and communication with healthcare professionals | Multiple chronic conditions | - | Adults |
| Rosenlund L et al., 2022 | PCC-IB | Recognition of patients as individuals and of their lived experiences; Active involvement in care planning; Bidirectional communication and partnership; Respect, empathy, and trust in healthcare professionals; Care coordination and continuity of care; Self-management support and identification of personal goals | Unspecified chronic conditions | - | Adults |
| Migchels C et al., 2024 | PREMAT-NL | Support in achieving personal goals; Improved self-esteem; Increased self-awareness; Privacy; Personal space; Understanding of substance use; Access to individual counselling sessions; Support for health, financial, and legal needs; Access to support for personal difficulties; Understanding of programme rules and expectations; Perceived appropriateness of programme rules; Structured daily routine; Support with time management; Opportunities for physical activity; Availability of healthy food options; Clean and hygienic care environment; Feeling supported and understood; Being treated as a person rather than as someone with substance use problems; Support for recovery; Family and friends' involvement in recovery; Improved ability to cope with daily life after the programme; Support with transitions to community services; Information about available support services | Mental health and substance use disorders | - | Adults |
| Haynes CJ et al., 2024 | CSQ-8; VSSS-MT; TPQ; HAQ; PCBSS | Internal, community, and healthcare-related stigma; Trust in healthcare professionals, compassion, respect for patients' preferences, safety, and communication; Perceived effectiveness of and engagement in treatment; Perceived control over medication dosing; Logistical and operational aspects of care; Physical and emotional side effects; Availability of women-specific treatment services; Financial barriers and insurance coverage; Accessibility of services and transportation; Fear of child protection involvement, loss of child custody, and employment or housing consequences; Pregnancy-related concerns and integration with maternal care services; Perceived impact of treatment on quality of life, craving reduction, and social and economic stability | Mental health and substance use disorders | - | Adults |
| Gasser F et al., 2023 | PCCoc | Welcoming care environment; Uninterrupted communication; Equal partnership in healthcare encounters; Being recognised as a person; Opportunity to share one's personal story; Understanding of patients' individual circumstances; Respect for patients' experiences; Self-awareness and self-understanding; Having concerns taken seriously; Care planning; Shared decision-making; Acquisition of new knowledge; Strengthening coping abilities; Care coordination; Family involvement; Comprehensive care documentation; Clarity of care responsibility; Sufficient time dedicated by healthcare professionals; Collaboration among healthcare professionals; Information sharing to facilitate care; Opportunity to influence care; Documentation of personal information; Appropriate use of personal information; Support for family members; Active participation in care; Encouragement to participate; Involvement in care; Participation in care-related decision-making; Participation in care planning | Multiple chronic conditions | - | Adults |
| Baart A et al., 2025 | CCQ; NCQ; PCI; CARE Measure; PCQ | Overall satisfaction with continuity of care; Perceived consistency of care; Perceived support during pregnancy and the postpartum period; Feeling of safety and trust in the primary healthcare professional; Active participation and involvement in decision-making; Readiness for parenthood; Effective communication and positive relationships with the care team | Maternal health | - | Adults |
| Howard FD et al., 2024 | MYCaW; consideRATE PREM; CSNAT; ASCOT-Carer | Perceived quality of life; Dignity; Autonomy; Relational support; Consistency and coordination of care | Palliative care | Frail older adults | Adults |
| Blake PG & Brown EA, 2020 | PAM | Active participation and involvement in care-related decision-making; Understanding and shared definition of care goals; Empathetic and transparent communication with the care team; Support from and trust in healthcare professionals; Education and informed choice regarding treatment options; Integration across healthcare services; Continuity, respect, and individualised care | Kidney disease | - | NR |
| Lloyd H et al., 2019 | P3CEQ | Person-centred care; Care coordination; Discussion of what matters most to patients; Shared decision-making; Recognition of patients as whole persons; Clear and consistent communication; Integrated care; Relational continuity with a designated healthcare professional; Shared care planning; Self-management support; Self-management information and education; Confidence in self-management | Multiple chronic conditions | - | Adults |
| Narayanan A et al., 2022 | DISQ | Feeling listened to and understood by the physician; Clear and comprehensive explanations; Reassurance and trust in the physician; Time dedicated during the consultation; Attention, respect, and courtesy; Empathy, compassion, and personal attention | - | General clinical population | Adults |
| Etingen B et al., 2016 | PAM; COMRADE; CARE Measure; PACIC; Press Ganey Outpatient Survey | Patient activation; Shared decision-making; Empathy and holistic care; Chronic disease management; Respect for patients' preferences; Overall experience with the Veterans Affairs (VA) healthcare system | - | Veterans | Adults |
| Eton DT et al., 2020 | PACIC | Treatment burden; Workload associated with treatment; Medication, appointments, and diagnostic test burden; Daily health management; Self-management routines; Impact on daily life; Physical and mental exhaustion; Financial burden; Access to and navigation of healthcare services; Medication side effects; Consideration of patients' social and cultural context; Care coordination and follow-up | Multiple chronic conditions | - | Adults |
| Willi D et al., 2025 | CAPE-CH | Need for improved communication and care coordination among healthcare professionals; Need for clearer information about treatment side effects and long-term consequences; Greater satisfaction with oncologist-led follow-up care; Experiences before diagnosis; Diagnostic pathway and communication of the diagnosis; Treatment decision-making; Outpatient and inpatient care; Home-based support and post-treatment follow-up; Overall satisfaction and patient feedback | Cancer | - | NR |
| Lyratzopoulos G et al., 2012 | GPPS | Communication with healthcare professionals; Access to care; Continuity of care; Overall satisfaction with care | - | General clinical population | Adults |
| Brito Fernandes Ó et al., 2020 | OECD PREMs | Waiting time for appointments; Waiting time at the healthcare facility; Time dedicated during the consultation; Clear and understandable explanations; Opportunity to ask questions and express concerns; Involvement in decision-making about care and treatment; Out-of-pocket payments | - | General clinical population | Adults |
| Piraux A et al., 2024 | PCCCT | Clarity of healthcare professionals' roles; Care coordination and continuity of care; Active involvement and recognition as partners in care; Empathy and effective communication within the healthcare team | Multiple chronic conditions | - | Adults |
| Eton DT et al., 2024 | PETS | Healthy lifestyle management; Financial burden of healthcare; Health monitoring; Medication management; Impact on personal relationships; Access to healthcare services; Health information | Multiple chronic conditions | Multiethnic groups | Adults |
| Barr PJ et al., 2017 | CollaboRATE | Information and explanations about health problems; Elicitation of patients' preferences; Integration of patients' preferences into decision-making | - | General clinical population | Adults |
| van Zwol-Janssens C et al., 2024 | Validated questionnaire | Overall care pathway experience; Perceptions of the multidisciplinary team; Relationships with healthcare professionals; Experience with patient-reported outcome and experience measures and questionnaire completion time; Waiting times; Appointment availability | Gynecological disorders | - | Adults |
| Haile ST et al., 2025 | QPP | Information about self-management; Knowing which nurse was responsible for care; Feeling respected by nurses; Feeling cared for by nurses; Ease of telephone access to the clinic; Opportunity to speak privately with the nurse; Difficulties contacting physicians by telephone; Difficulties scheduling appointments with physicians | Cardiovascular diseases | - | Older adults |
| Cunningham R et al., 2024 | PCPES | Relationships with healthcare professionals; Barriers to access; Fulfilment of individual and mental health needs | - | General clinical population | Mixed |
| Marsall M et al., 2024 | CTM-19 | Critical understanding of health information; Preparedness for self-management; Care planning; Patients' important preferences | - | General clinical population | Adults |
| Strohschein FJ et al., 2024 | AOPSS | Respect for patients' preferences; Physical comfort; Access to care; Care coordination and continuity of care; Information and communication; Emotional support | Cancer | - | Adults |
| Christalle E et al., 2025 | EPAT-16 | Healthcare professionals' sensitivity, empathy, and understanding; Trusting clinician–patient relationships; Recognition of patients as unique individuals; Biopsychosocial approach to care; Clinician–patient communication; Integration of medical and non-medical care; Teamwork and care coordination; Access to healthcare professionals; Care coordination and continuity of care; Patient safety; Clear and comprehensive health information; Patient involvement in care-related decision-making; Family and friends' involvement in care; Patient empowerment; Timely physical support | - | General clinical population | Adults |
| Hoepner JK et al., 2024 | mCARE-TBI | Appropriateness of acute care; Hospital-to-home transition; Clear discharge information; Family involvement in care; Active listening; Compassionate and caring approach; Positive attitude; Recognition of the consequences of traumatic brain injury; Management of symptoms and patients' concerns; Collaboration with patients | Neurological disorders | - | Adults |
| Ho L et al., 2013 | CAHPS | Access to care; Physician–patient communication; Support from healthcare staff; Physician's knowledge of the patient | - | General clinical population | Adults |
| Bonney et al., 2022 | PCAT-S | Relational continuity; Care coordination; Accessibility of care; Comprehensiveness of care; Self-perceived health | Multiple chronic conditions | - | Adults |
| Chung et al., 2020 | CG-CAHPS | Clear and understandable explanations; Active listening by the healthcare provider; Respectful communication; Time dedicated during the consultation; Timely access to urgent care; Timely access to routine care; Timely responses to medical questions; Overall provider rating | - | General clinical population | Adults |
| Gibson et al., 2025 | GPPS | Overall experience with general practice; Experience with appointment access | - | General clinical population | NR |
| Norman et al., 2024 | PEQ-GP | Accessibility of care; Communication and interpersonal aspects of care; Continuity of care; Professionalism of healthcare professionals; Organisational aspects of care | - | General clinical population | Adults |
| Medina-Ranilla et al., 2023 | National Health Survey – Brazil (Pesquisa Nacional de Saúde, PNS); National Quality of Life Survey – Colombia (Encuesta Nacional de Calidad de Vida); ENSANUT; IMSS Patient Exp. Survey | Overall satisfaction; Waiting times; Clear communication; Continuity of care; Privacy; Dignity; Equity and non-discriminatory care; Trust in healthcare professionals; Resolution of health problems; Involvement in decision-making | - | General clinical population | Adults |
| Stynes et al., 2021 | FFT; PEI; CollaboRATE; Valuing Patients as Individuals | Perceived quality of the physiotherapist–patient relationship and physiotherapist competence; Effective communication, active listening, and patient involvement; Active participation in treatment decision-making; Greater empowerment in the self-management of musculoskeletal conditions; Overall satisfaction with and willingness to recommend the service | Musculoskeletal disorders | - | Adults |
| Nadeau et al., 2024 | APEX Q. | Access to care; Waiting times; Experience with reception staff; Cleanliness of the healthcare facility; Comfort of the waiting area; Time dedicated by healthcare professionals; Attention to patients' needs and preferences; Respectful and dignified treatment; Overall experience with physicians and nurses; Overall experience with care; Willingness to recommend the service; Clear and understandable information; Self-management support; Perceived severity of illness; Self-perceived physical and mental health | Respiratory diseases | - | Adults |
| Alasqah, 2023 | PREOS-PC | Perceived problems with diagnosis; Healthcare professional–patient communication; Medication errors; Vaccine safety; Diagnostic testing; Trust in healthcare professionals and perceived commitment to patient safety | - | General clinical population | Adults |
| Fiol-deRoque et al., 2024 | PREOS-PC-Compact | Patient safety in primary care; Perceived diagnostic, medication, and treatment errors; Frequency of perceived safety incidents; Communication and relationship with the primary care physician; Feeling listened to and taken seriously; Clear explanations of diagnoses and treatments; Trust in the primary care physician; Patient involvement in safety; Promotion of patient participation in error prevention; Perceived consequences of safety incidents; Physical, emotional, and financial impact of safety incidents; Impact on trust in the healthcare system; Overall perception of patient safety | - | General clinical population | Mixed |
| Thomas et al., 2024 | PREOS-PC | Trust in the healthcare system; Preference for face-to-face consultations; Difficulties accessing appointments; Value placed on timely diagnosis, clear communication, and individualised care | Musculoskeletal disorders | - | Adults |
| Serrano-Ripoll, Fiol-Deroque, et al., 2022 | PREOS-PC; MOSPSC | Patients' experiences of safety; Experienced harm events; Patient activation and involvement; Evaluations of general practice performance | - | General clinical population | NR |
| O’Keeffe et al., 2021 | Experiences of Health and Social Care Q. | Long delays in diagnosis and referral; Feeling disrespected and stigmatised by physicians and general practitioners; Lack of care coordination and limited involvement in care-related decision-making; Greater negative financial impact | Neurological disorders | - | Adults |
| d’Agincourt-Canning et al., 2024 | Hua Oranga; Native Wellness Assessment; Cultural Safety Survey; Cultural Connectedness Scale; EOHCS; Awareness of Connectedness Scale; GEM; Multicultural Mastery Scale; Indigenous Peoples' Survey | Trusting relationships; Ethical spaces for care; Ceremonies and storytelling; Holistic approach to health; Experiences of racism; Family and community involvement; Strengths and resilience | - | Indigenous population | NR |
| Siskou et al., 2025 | PaRIS Survey | Accessibility of care; Care coordination; Physician–patient communication; Continuity of care | - | General clinical population | Adults |
| Nasser Albarqi, 2024 | UPC; COCI | Satisfaction with continuity of care; Trust in the relationship with the primary care physician; Perceived care coordination and support for chronic disease management; Perceived safety during care transitions | Multiple chronic conditions | - | Adults |
| Maroni et al., 2022 | IAPS | Welcoming environment and preparedness; Clarity of information; Comfort and perceived discomfort; Professionalism and attitude of healthcare professionals; Overall evaluation of the procedure | Gastrointestinal disorders | - | Adults |
| Ryan et al., 2025 | Eye-Care PREM | Geographical accessibility and reduced logistical burden; Clear and essential clinical communication; Greater trust through clear and continuous information; Frustration and anxiety due to communication delays and lack of transparency | Ophthalmologic disorders | - | Adults |
| Fiol-deRoque et al., 2025 | PREOS-PC-Compact | Healthcare practice engagement in patient safety; Patient engagement in safety; Experiences of perceived patient safety problems in primary care; Perceived severity of harm; Impact of harm on daily life; Overall perception of patient safety | - | General clinical population | Mixed |
| Kutyla et al., 2023 | Comprehensive Endoscopy Satisfaction Tool (CEST) | Clarity and completeness of information provided; Comfort during the procedure; Waiting times before and during the endoscopic procedure; Support from healthcare professionals during preparation; Quality of post-procedure care, including recovery and discharge instructions; Healthcare facility environment; Overall satisfaction and willingness to recommend the service (Net Promoter Score) | Postoperative conditions | - | Adults |
| Valderas et al., 2024 | PaRIS Survey | Access to care; Comprehensiveness and individualised care; Continuity of care; Care coordination; Perceived patient safety; Self-management support; Trust in healthcare professionals; Overall quality of care | Multiple chronic conditions | - | Adults |
| Barello et al., 2021 | WE-CARE IBD Score | Access to high-quality professional and clinical care; Individualised care planning; Continuity of care and ongoing information; Being treated as a person rather than just a patient; Quality of communication and therapeutic relationships | Gastrointestinal disorders | - | Adults |
| Torresdey et al., 2024 | CG-CAHPS | Understanding of information provided by the clinician; Feeling listened to and respected by the clinician; Interpreter support during communication; Navigation of the care pathway; Guidance regarding follow-up care and treatment | - | Population with language barriers | Adults |
| Burgess et al., 2023 | MSK PREM | Respectful and compassionate care; Feeling understood and valued; Trust in healthcare professionals' clinical competence; Shared decision-making; Time dedicated by healthcare professionals; Perceived overall improvement in health; Improved understanding of one's health condition; Greater confidence in self-management; Timeliness and convenience of consultations; Access to musculoskeletal (MSK) care and appointment availability | Musculoskeletal disorders | - | Adults |
| Ruiz et al., 2025 | CG-CAHPS | Active listening by the physician; Respectful communication; Encouragement to ask questions; Time dedicated during the consultation; Interpreter support in expressing patients' concerns; Interpreter support in understanding clinical instructions; Courteous and respectful treatment by the interpreter; Overall experience with the interpreter | - | Population with language barriers | Adults |
| de Camargo Silva et al., 2024 | PREOS-PC-Compact Brazil | Healthcare practice engagement in patient safety; Patient engagement in safety; Experiences of patient safety incidents; Physical, psychological, social, and financial impact of safety incidents; Overall perception of patient safety | - | General clinical population | Adults |
| Paddison et al., 2018 | GPPS | Trust and confidence in healthcare professionals; Quality of communication with healthcare professionals; Time dedicated during the consultation; Active listening; Explanations about diagnostic tests and treatments; Involvement in care-related decision-making; Compassionate and caring treatment | - | General clinical population | Mixed |
| Grønhaug et al., 2014 | OA-QI | Information about the disease and its progression; Information about treatment options; Self-management education; Lifestyle advice; Information about the importance of physical activity and exercise; Referral to physical activity programmes; Weight management advice; Referral for weight management support; Functional assessment; Assessment of walking aid needs; Assessment of other assistive devices; Pain assessment; Information about paracetamol use; Information about stronger analgesics; Advice on non-steroidal anti-inflammatory drugs (NSAIDs); Corticosteroid injections when appropriate; Referral to an orthopaedic specialist when indicated | Musculoskeletal disorders | - | Adults |
| Campbell et al., 2020 | PREOS-PC | Difficulties accessing care and scheduling appointments; Problems with the diagnostic process; Inadequate patient–physician communication; Poor coordination among healthcare professionals within the same practice; Lack of care coordination across healthcare settings; Problems related to medication management | - | General clinical population | Adults |
| Valderas et al., 2019 | NR | Continuity and care coordination; Patient-centred care and involvement in decision-making; Experiences of fragmented care and critical care transitions; Satisfaction with care received | Multiple chronic conditions | - | Adults |
| Doubova et al., 2020 | PCQCCQ | Timely care; Clear information; Information to support treatment decision-making; Attention to patients' biopsychosocial needs; Respectful and coordinated care | Cancer | - | Adults |
| Glenngård & Anell, 2018 | NPE | Accessibility of care; Relational continuity with healthcare professionals | - | General clinical population | Adults |
| Cohidon et al., 2018 | QUALICOPC | Access to care; Physician–patient communication; Relational continuity and care coordination | - | General clinical population | Adults |
| Schäfer et al., 2019 | QUALICOPC | Communication, access to care, continuity of care, comprehensiveness of care, and involvement in decision-making; Patients' perceived potential for improvement in care; Equity of care; Inefficient use of healthcare services; Patient enablement | - | General clinical population | Adults |
| Solheim et al., 2017 | PasOpp Patient Exp. Q. | Overall satisfaction with care received; Trust in the physician's professional competence; Clear and understandable communication; Expectation of receiving high-quality hospital care; Opportunity to discuss what matters most to patients; Involvement in treatment decision-making; Perceived inadequacy of information about the disease and treatment; Insufficient frequency of follow-up visits; Delays in appointments | Neurological disorders | - | Adults |
| Ricci-Cabello, Saletti-Cuesta, et al., 2017 | PREOS-PC | Difficulties accessing care and scheduling appointments; Poor communication with administrative staff; Lack of relational continuity with healthcare professionals; Inadequate patient–physician communication; Lack of active listening, empathy, and time dedicated by healthcare professionals; Feeling dismissed or judged; Medication errors; Diagnostic errors and delays; Communication of diagnostic test results; Follow-up of care; Accuracy of medical records; Care coordination across healthcare professionals and services; Hospital-to-community care transitions; Emotional impact of care experiences; Trust in healthcare professionals and the healthcare system; Active patient involvement in managing care and safety; Patients' recommendations for improving care quality and patient safety | Internal medicine conditions | - | Adults |
| Zulman et al., 2019 | SHEP; PACIC; Health Care Hassles Scale | Access to care and communication with the care team; Care coordination and management of chronic conditions; Perceived barriers and challenges in the care pathway; Trusting relationship with the healthcare provider; Consideration of patients' health goals and barriers by the care team; Overall and service-specific satisfaction with primary care | - | Veterans | Adults |
| McDonald et al., 2018 | PACIC-11; CollaboRATE; PAM | Overall experience with chronic illness care; Self-management support; Continuity and care coordination; Collaboration and involvement in care-related decision-making; Perceived support tailored to patients' needs; Involvement of the multidisciplinary team in chronic care management | Multiple chronic conditions | - | Adults |
| (Wallace et al., 2010 | PACIC | Patient activation; Organisation and clarity of care; Shared goal setting; Problem-solving support and contextual counselling; Continuity and care coordination; Clear and understandable communication; Active involvement in decision-making; Ongoing practical support; Care coordination among healthcare professionals | Metabolic disorders | - | Adults |
| Aysola et al., 2018 | Press Ganey Outpatient Survey | Ease of contacting the general practice; Courtesy and helpfulness of administrative staff; Clarity and timeliness of information; Waiting times and communication about delays; Courtesy of nursing staff; Overall organisation of care; Clarity of medical explanations; Attention to patients' concerns; Involvement in treatment decision-making; Time dedicated during the consultation and trust in healthcare professionals; Cleanliness and hygiene of the healthcare facility; Respect for privacy; Sensitivity to individual needs; Preventive care reminders; Management of comorbidities; Communication of test results and information sharing among healthcare professionals | Internal medicine conditions | - | Adults |
| Wong et al., 2019 | SHEP | Access to care; Timely access to specialist care and diagnostic services; Listening, respect, and time dedicated by healthcare professionals; Clear communication and involvement in care; Pharmacy services; Organisation of care and healthcare facility environment; Overall experience with care; Trust in the healthcare system | - | Veterans | Older adults |
| Ricci-Cabello, Marsden, et al., 2017 | PREOS-PC | Access to and continuity of care; Communication and relationships with healthcare professionals; Care coordination among healthcare professionals; Experiences of patient safety incidents; Perceived harm and healthcare system response | - | General clinical population | Adults |
| Altin & Stock, 2015 | IHP Survey | Timely access to care; Support with care coordination; Overall satisfaction with primary care | - | General clinical population | Adults |
| Ricci-Cabello, Reeves, et al., 2017 | PREOS-PC | Experiences of patient safety problems in primary care; Experiences of healthcare-related harm; Overall perception of patient safety | - | General clinical population | Adults |
| Crocker et al., 2017 | CAHPS; ACES; CARE Measure | Whole-person care; Health promotion, well-being, and self-management support; Quality of relationships and communication with healthcare professionals; Access to care; Overall satisfaction with care received | - | General clinical population | Adults |
| Ricci-Cabello et al., 2016 | PREOS-PC | Experiences of patient safety problems; Experiences of healthcare-related harm; Overall perception of patient safety; Patient and healthcare practice engagement in risk prevention | - | General clinical population | Adults |
| Ricci-Cabello et al., 2021 | PREOS-PC | Patient–physician communication problems; Inadequate care coordination among healthcare professionals; Difficulties accessing appointments; Problems with laboratory testing; Delayed or inappropriate treatment; Physical, psychological, and social impact of healthcare-related harm | - | General clinical population | Adults |
| Serrano-Ripoll, Llobera, et al., 2022 | PREOS-PC-Compact | Availability of the primary care physician; Feeling that health concerns are taken seriously; Information about medication side effects; Encouragement to discuss concerns about care; Patient engagement in identifying safety problems; Patient engagement in suggesting improvements to care; Experiences of patient safety incidents involving diagnosis, medications, treatments, vaccinations, laboratory testing, diagnostic procedures, communication with healthcare professionals, care coordination, appointments, and medical records | - | General clinical population | Mixed |
| Mounce et al., 2022 | PREOS-PC; PREOS-PC-Compact; PREOS-PC-Screen | Availability of the primary care physician; Feeling that health concerns are taken seriously; Encouragement to ask questions; Information about medication side effects; Patient engagement in reporting safety concerns; Patient engagement in suggesting improvements; Diagnostic problems; Medication-related problems; Problems with other treatments; Vaccine-related problems; Problems with blood and laboratory tests; Problems with other diagnostic procedures; Patient–healthcare professional communication; Communication among healthcare professionals; Communication across healthcare settings; Appointment-related problems; Medical record-related problems | - | General clinical population | Adults |
| Booker et al., 2025 | PREOS-PC | Timely access to care; Reassurance regarding paramedics' competence and patient safety; Communication with paramedics; Feeling listened to and treated with respect | - | General clinical population | NR |
| Wang et al., 2025 | PCPCM | Accessibility of care; Comprehensiveness of care; Integration of care; Care coordination; Relational continuity with the primary care physician; Continuity of care; Patient advocacy; Consideration of the family context; Consideration of the community context; Health promotion; Goal-oriented care | - | General clinical population | Adults |
| Jelin et al., 2024 | Validated questionnaire | Waiting times for urgent and routine appointments; Waiting times at the healthcare facility; Delayed or missed referrals; Insufficient consultation time; Difficulties contacting the general practice by telephone; Problems with digital communication and appointment booking; Geographical accessibility of the primary care physician; Disrupted continuity of care; Feeling that concerns are not given sufficient attention or respect by the primary care physician | - | General clinical population | Adults |
| Hasnain et al., 2013 | CG-CAHPS | Overall provider rating; Willingness to recommend the provider; Shared decision-making; Provider communication; Communication among healthcare professionals; Evaluation of other healthcare providers; Support and courtesy from healthcare staff; Timely access to appointments | - | General clinical population | Adults |
| Bradley et al., 2020 | OPTIQ | Comfort during orthodontic procedures; Perceived treatment-related complications; Impact of treatment on daily life; Recall of information provided about treatment and self-care | Postoperative conditions | - | Adults |
| Philpot et al., 2019 | Press Ganey Medical Practice Survey | Access to care; Communication with the healthcare provider; Overall experience with care; Willingness to recommend the service | Internal medicine conditions | - | Adults |

# Color legend

Instrument classification

|  | **Validated PREMs only** Studies using exclusively standardized and validated PREMs. |
| --- | --- |
|  | **Validated PREMs + ad hoc instruments** Studies using both standardized validated PREMs and study-specific ad hoc instruments. |

Pathology

|  | Intellectual disability |  | Cancer |
| --- | --- | --- | --- |
|  | Hearing disorders |  | Dermatological disorders |
|  | Mental health and substance use disorders |  | Metabolic disorders |
|  | Neurological disorders |  | Respiratory diseases |
|  | Musculoskeletal disorders |  | Palliative care |
|  | Cardiovascular diseases |  | Gynecological disorders |
|  | Unspecified chronic conditions |  | Gastrointestinal disorders |
|  | Multiple chronic conditions |  | Ophthalmologic disorders |
|  | Maternal health |  | Postoperative conditions |
|  | Kidney disease |  | Internal medicine conditions |

Age group

|  | Pediatric |
| --- | --- |
|  | Adults |
|  | Mixed |
|  | Older adults |
|  | NR |

Population

|  | General clinical population |
| --- | --- |
|  | Vulnerable population |
|  | Frail older adults |
|  | Veterans |
|  | Population with language barriers |
|  | Indigenous population |
|  | Multiethnic groups |

*Note. White cells indicate categories that were not applicable or were not reported.*

# PREMs instruments legend

| **Acronym** | **Full instrument name** | **Acronym** | **Full instrument name** |
| --- | --- | --- | --- |
| AASPIRE Survey | AASPIRE Healthcare Experiences Survey; | JPCAT-SF | Japanese Primary Care Assessment Tool – Short Form |
| P3CEQ | Person-Centred Coordinated Care Experience Questionnaire | CAHPS | Consumer Assessment of Healthcare Providers and Systems |
| UK Audiology CQI | UK Audiology Care Questionnaire | NHS Patient Survey | NHS Patient Survey |
| SCQ | Sinclair Compassion Questionnaire | PEDS | Patient Experience of Diabetes Service Survey (PEDS) |
| EQUIP-PH-PREM | EQUIP Physical Health PREM | GPPS | GP Patient Survey |
| SAATSA | South African Addiction Treatment Services Assessment | PCCQ | Patient Continuity of Care Questionnaire |
| COPES | Community Oriented Programs Environment Scale | DICARES | Discharge Care Experiences Survey |
| PCAS | Primary Care Assessment Survey | B-PREPARED | B-PREPARED Discharge Readiness Scale |
| NQR-SAT/KVARUS | National Quality Register for Substance Abuse Treatment | PACT-M | Partners at Care Transitions Measure |
| ECHO | Experiences of Care and Health Outcomes Survey | CTM | Care Transitions Measure (CTM) |
| EOHCS-Ongoing | Equity-Oriented Health Care Scale – Ongoing | MSK Indicators | Musculoskeletal Care Indicators |
| EOHCS-Episodic | Equity-Oriented Health Care Scale – Episodic | DREMS | Diabetes Reported Experience Measure Survey |
| PREMAT | Patient Reported Experience Measure for Addiction Treatment | Inpatient Diabetes PREM (Inpatient Diabetes PREM) | Inpatient Diabetes Patient Reported Experience Measure (Inpatient Diabetes PREM) |
| Experiences of Patient-Centred Care Q | Experiences of Patient-Centred Care Questionnaire | EDP Questionnaire | EDP Diabetes Patient Experience Questionnaire |
| AOD Experience Questionnaire | Alcohol and Other Drug Experience Questionnaire | Outpatient Clinic PREM | Outpatient Clinic Patient Reported Experience Measure (Outpatient Clinic PREM) |
| CPC | Client Perception of Care | APEQ-DC | Adolescent Patient Experience Questionnaire for Diabetes Care |
| SUSOS | Service Users’ Satisfaction and Outcomes Survey | NDR PREM | Swedish National Diabetes Register Patient Experience Survey |
| PEQ-ITSD | Patient Experience Questionnaire for Interdisciplinary Treatment for Substance Dependence | T1D Youth PREM | Type 1 Diabetes Youth Patient Reported Experience Measure |
| Epilepsy12 PREM | Epilepsy12 Patient/Carer and Child Experience Questionnaire | CSAT | Customer Satisfaction Score |
| Dutch Hospital Care PREM | PREM Ziekenhuiszorg | KB Patient Experience Survey | Kootenay Boundary Patient Experience Survey |
| KHPS | Korea Health Panel Survey | PREOS-PC | Patient-Reported Experiences and Outcomes of Safety in Primary Care |
| CANHELP Lite (Patient) | CANHELP Lite – Patient Version | PREM-CC | Chronic Care Patient Reported Experience Measure |
| OARS CSQ | OARS Client Satisfaction Questionnaire | PREM CatSalut | CatSalut Primary Care Patient Experience Survey |
| CANHELP Lite (Family) | CANHELP Lite – Family Version | PCC-IB | Person-Centred Care Item Bank |
| NCQ | Nijmegen Continuity Questionnaire | PREMAT-NL | Patient Reported Experience Measure in Addiction Treatment |
| PECQ | Patient Experienced Continuity of Care Questionnaire | CSQ-8; | Client Satisfaction Questionnaire |
| PEQ-PC | Patient Experience Questionnaire in Primary Care | PCBSS | Primary Care Buprenorphine Satisfaction Scale |
| VSQ-9 | Visit-Specific Satisfaction Questionnaire | VSSS-MT | Verona Service Satisfaction Scale for Methadone Treatment |
| PCAT | Primary Care Assessment Tool | PCCoc | Person-Centred Care Instrument for Outpatient Care |
| CSQ-8 | Client Satisfaction Questionnaire | CCQ | Continuity of Care Questionnaire |
| PEI | Patient Enablement Instrument | PCQ | Pregnancy and Childbirth Questionnaire |
| NPIS | Nurse–Patient Interaction Scale | PCI | Patient Continuity Index |
| QPP | Quality of Care from the Patient’s Perspective | CSNAT | Carer Support Needs Assessment Tool |
| GPAQ | General Practice Assessment Questionnaire | consideRATE PREM | consideRATE Patient Experience Measure |
| PSQ | Patient Satisfaction Questionnaire | ASCOT-Carer | Adult Social Care Outcomes Toolkit for Carers |
| FFT | Friends and Family Test | MYCaW; | Measure Yourself Concerns and Wellbeing |
| PTE | Patient-PTE Questionnaire | DISQ | Doctor’s Interpersonal Skills Questionnaire |
| PreMaPEQ | Pregnancy and Maternity Care Patients’ Experiences Questionnaire | COMRADE | Combined Outcome Measure for Risk Communication and Treatment Decision Making |
| Home-DCE | Home Dialysis Care Experience Instrument | CAPE-CH | Cancer Patient Experience Questionnaire – Switzerland |
| Kidney PREM-SF | Kidney Patient Reported Experience Measure – Short Form | OECD PREMs | OECD Standardised Patient-Reported Experience Measures |
| Needling PREM | Needling Patient Reported Experience Measure | PCCCT | Patient-Centered Coordination by a Care Team |
| Pediatric Kidney PREM | Pediatric Kidney Patient Reported Experience Measure | PETS | Patient Experience with Treatment and Self-Management |
| Kidney PREM | Kidney Patient Reported Experience Measure | QPP | Quality from the Patient’s Perspective |
| ICH CAHPS | In-Center Hemodialysis Consumer Assessment of Healthcare Providers and Systems | PCPES | Primary Care Patient Experience Survey |
| CCAENA | Continuity of Care Across Levels Questionnaire | CTM-19 | Care Transitions Measure |
| PCCQ | Patient Continuity of Care Questionnaire | AOPSS | Ambulatory Oncology Patient Satisfaction Survey |
| ACS | Alberta Continuity of Care Survey | EPAT-16 | Experience of Patient Assessment Tool – Short Form |
| Chao | Patient-Perceived Continuity of Care Scale | mCARE-TBI | Consultation and Relational Empathy Measure – TBI version |
| CCI | Care Continuity Index | PCAT-S | Primary Care Assessment Tool – Short Form |
| PACIC | Patient Assessment of Chronic Illness Care | PEQ-GP | Patient Experience Questionnaire for General Practice |
| ACES-SF | Ambulatory Care Experiences Survey – Short Form | ENSANUT | National Health and Nutrition Survey – Mexico |
| CPCI | Components of Primary Care Index | IMSS Patient Exp. Survey | IMSS Patient Experience Survey |
| SHEP | Survey of Healthcare Experiences of Patients | National Health Survey – Brazil (Pesquisa Nacional de Saúde, PNS) | National Health Survey – Brazil (Pesquisa Nacional de Saúde, PNS |
| PPEQ | Picker Patient Experience Questionnaire | APEX Q. | APEX Questionnaire |
| Press Ganey Patient Exp. Survey | Press Ganey Patient Experience Survey | PREOS-PC-Compact | Patient-Reported Experiences and Outcomes of Safety in Primary Care – Compact |
| PAM | Patient Activation Measure | MOSPSC | Medical Office Survey on Patient Safety Culture |
| DDSM-QM | Diabetes Disease State Management Questionnaire – Modified | Experiences of Health and Social Care Q. | Experiences of Health and Social Care Questionnaire |
| CG-CAHPS | Clinician & Group Consumer Assessment of Healthcare Providers and Systems | EOHCS | Equity-Oriented Health Care Scale |
| IC-PREMs (Bed-based) | Intermediate Care Patient Reported Experience Measures – Bed-based | GEM | Growth and Empowerment Measure |
| IC-PREMs (Home-based) | Intermediate Care Patient Reported Experience Measures – Home-based | Hua Oranga; Native Wellness Assessment | Hua Oranga; Native Wellness Assessment |
| Press Ganey Outpatient Survey | Press Ganey Outpatient Medical Practice Survey | PaRIS Survey | OECD Patient-Reported Indicator Surveys |
| CEST | Client Evaluation of Self and Treatment | COCI | Continuity of Care Index |
| PIS | Patient Involvement Scales | UPC | Usual Provider of Care Index |
| HAQ | Helping Alliance Questionnaire | IAPS | Inventory to Assess Patient Satisfaction |
| TPQ | Treatment Perceptions Questionnaire | Eye-Care PREM | Eye-Care Patient Reported Experience Measure |
| TCU-CEF | Texas Christian University Client Evaluation Form | CEST | Comprehensive Endoscopy Satisfaction Tool |
| EORTC QLQ-INFO26 | EORTC Quality of Life Questionnaire – Information Module | MSK PREM | National Musculoskeletal Patient Reported Experience Measure |
| NPS | Net Promoter Score | PREOS-PC-Compact Brazil | Patient-Reported Experiences and Outcomes of Safety in Primary Care – Compact |
| Home-DCE | Home Dialysis Care Experience | OA-QI | Osteoarthritis Quality Indicator Questionnaire |
| Home Dialysis Center Care and Operations Scale | Quality of Home Dialysis Center Care and Operations Scale | PCQCCQ | Patient-Centered Quality of Cancer Care Questionnaire |
| PCQ-PD | Parkinson's Disease Care Questionnaire | NPE | National Patient Experience Survey |
| PQRS | Patient Questionnaire for Rehabilitation Services | QUALICOPC | Quality and Costs of Primary Care Questionnaire |
| CARE Measure | Consultation and Relational Empathy Measure | PasOpp Patient Exp. Q. | PasOpp Patient Experience Questionnaire |
| PSQ-18 | Patient Satisfaction Questionnaire Short Form | PACIC-11 | Patient Assessment of Chronic Illness Care – Short Form |
| OPEQ | Outpatient Experience Questionnaire | IHP Survey | Commonwealth Fund International Health Policy Survey |
| QUOTE-EEE | Quality of Care from the Patient's Perspective | ACES | Ambulatory Care Experiences Survey |
| HINTS | Health Information National Trends Survey | PREOS-PC-Screen | Patient-Reported Experiences and Outcomes of Safety in Primary Care – Screen |
| ECHO | Experience of Care and Health Outcomes | PCPCM | Primary Care Patient-Centered Measure |
| PCQRS | Patient Care Questionnaire for Rehabilitation Services | OPTIQ | Orthodontic Patient Treatment Impact Questionnaire |
| EUROPEP | European Task Force on Patient Evaluations of General Practice Care |  |  |
