# Supplementary material for: Patient-reported experiences and patient-reported experience measures in primary care, home care, and community settings. A scoping review
Source: Front Rehabil Sci. 2026 Sep 24;7:1966101. doi: 10.3389/fresc.2026.1966101 (PMC13646105; doi:10.3389/fresc.2026.1966101)
Supplement: Supplementary file 4 [file Table4.docx]

| **Table S4.** PRE categories |  |  |
| --- | --- | --- |
| **PRE category (references)** | **Representative content** | **Frequency (n)** |
| **Communication and information** (Ahern et al., 2019; Arnold et al., 2022; Bajgain et al., 2020; Baker et al., 2015; Bryant et al., 2023; Coelho et al., 2022; Deville-Stoetzel N et al., 2024; Frahsa et al., 2020; Fu et al., 2023; Giannopoulos E et al., 2024; Goldthorpe J et al., 2018; Hopstaken et al., 2021; Humphries et al., 2021; Jackson et al., 2019; Krist et al., 2008; Lamprell K et al., 2023; Laws et al., 2019; C. Lin et al., 2022; E. Lin et al., 2022; Luckett T et al., 2022; McErlane F et al., 2018; Montgomery-Taylor S et al., 2016; Nuti S et al., 2017; Price et al., 2022; Rodríguez-Fuertes et al., 2023; Savla et al., 2025; Tou et al., 2022; Vaitkeviciene et al., 2023; van der Sar et al., 2021; Walker et al., 2017; Williams et al., 2018; Zeh S et al., 2019; Zigman Suchsland ML et al., 2019) | Communication with healthcare professionals; clear, complete and consistent information; health education; opportunity to ask questions; understanding of diagnosis, treatment and care pathway. | 62 |
| **Access to services** (Abelsen et al., 2023; Ahern et al., 2019; Bajgain et al., 2020; Breton et al., 2024; Bryant et al., 2023; Deville-Stoetzel N et al., 2024; Frahsa et al., 2020; Fu et al., 2023; Giannopoulos E et al., 2024; Goldthorpe J et al., 2018; F. Graham et al., 2021; Hopstaken et al., 2021; Howard AF et al., 2024; Huhtanen et al., 2025; C. Lin et al., 2022; Luckett T et al., 2022; McErlane F et al., 2018; Montgomery-Taylor S et al., 2016; Nuti S et al., 2017; Olding et al., 2018; Pourmarzi et al., 2019; Price et al., 2022; Savla et al., 2025; Tacchini-Jacquier N et al., 2024; Tou et al., 2022; Vaitkeviciene et al., 2023; van der Sar et al., 2021; White TM et al., 2024; Williams et al., 2018; Zeh S et al., 2019) | Access to healthcare services; appointment availability; waiting times; accessibility; contact with healthcare providers; barriers to access. | 51 |
| **Patient–professional relationship** (Ahern et al., 2019; Bryant et al., 2023; Coelho et al., 2022; Frahsa et al., 2020; Fu et al., 2023; Haex R et al., 2020; Howard AF et al., 2024; Huhtanen et al., 2025; Jackson et al., 2019; Lamprell K et al., 2023; C. Lin et al., 2022; E. Lin et al., 2022; Luckett T et al., 2022; McErlane F et al., 2018; Montgomery-Taylor S et al., 2016; Nuti S et al., 2017; Pourmarzi et al., 2019; Rodríguez-Fuertes et al., 2023; Tou et al., 2022; Vaitkeviciene et al., 2023; Walker et al., 2017; White TM et al., 2024; Zeh S et al., 2019; Zigman Suchsland ML et al., 2019) | Relationship with healthcare professionals; trust, empathy and respect; active listening; supportive and patient-centred care. | 43 |
| **Quality of care** (Abelsen et al., 2023; Ahern et al., 2019; Baker et al., 2015; Bryant et al., 2023; Cowell et al., 2024; Goldthorpe J et al., 2018; Huhtanen et al., 2025; Jackson et al., 2019; Lamprell K et al., 2023; C. Lin et al., 2022; E. Lin et al., 2022; Luckett T et al., 2022; Nuti S et al., 2017; Price et al., 2022; Rodríguez-Fuertes et al., 2023; Tacchini-Jacquier N et al., 2024; Tou et al., 2022; Walker et al., 2017; Zeh S et al., 2019) | Perceived quality, effectiveness and benefits of healthcare services and treatments. | 38 |
| **Service organization** (Abelsen et al., 2023; Bryant et al., 2023; Caneiras C et al., 2022; Coelho et al., 2022; Deville-Stoetzel N et al., 2024; Fu et al., 2023; Goldthorpe J et al., 2018; Howard AF et al., 2024; Huhtanen et al., 2025; Lamprell K et al., 2023; Luckett T et al., 2022; McErlane F et al., 2018; Nuti S et al., 2017; Rodríguez-Fuertes et al., 2023; Vaitkeviciene et al., 2023; Walker et al., 2017; White TM et al., 2024; Williams et al., 2018; Zeh S et al., 2019; Zigman Suchsland ML et al., 2019) | Service organization; care pathways; care planning; coordination and continuity; treatment management; patient participation; service improvement. | 34 |
| **Continuity of care** (Abelsen et al., 2023, 2023; Arnold et al., 2022; Baker et al., 2015; Breton et al., 2024; Bryant et al., 2023; Deville-Stoetzel N et al., 2024; Giannopoulos E et al., 2024; Haex R et al., 2020; Hopstaken et al., 2021; Howard AF et al., 2024; Jackson et al., 2019; Luckett T et al., 2022; Nuti S et al., 2017; Tacchini-Jacquier N et al., 2024; Vaitkeviciene et al., 2023; Williams et al., 2018) | Continuity of care; care coordination; follow-up and monitoring; care transitions; integrated and ongoing care. | 24 |
| **Emotional experience** (Ahern et al., 2019; Baker et al., 2015; Caneiras C et al., 2022; Giannopoulos E et al., 2024; J. Graham et al., 2007; Howard AF et al., 2024; Lamprell K et al., 2023; Laws et al., 2019; Luckett T et al., 2022; Rodríguez-Fuertes et al., 2023; Savla et al., 2025; Tou et al., 2022; Vaitkeviciene et al., 2023; van der Sar et al., 2021; Walker et al., 2017; White TM et al., 2024; Zeh S et al., 2019; Zigman Suchsland ML et al., 2019) | Emotional and psychological impact; emotional support; anxiety, fear and frustration; coping; social impact. | 23 |
| **Patient involvement** (Arnold et al., 2022; Bryant et al., 2023; Caneiras C et al., 2022; Coelho et al., 2022; Fu et al., 2023; Haex R et al., 2020; Hopstaken et al., 2021; Howard AF et al., 2024; Jackson et al., 2019; Krist et al., 2008; Lamprell K et al., 2023; C. Lin et al., 2022; Luckett T et al., 2022; Montgomery-Taylor S et al., 2016; Savla et al., 2025; White TM et al., 2024; Zeh S et al., 2019) | Patient involvement and participation; shared decision-making; patient empowerment and autonomy. | 20 |
| **Care coordination** (Abelsen et al., 2023; Arnold et al., 2022; Bryant et al., 2023; Deville-Stoetzel N et al., 2024; Giannopoulos E et al., 2024; Howard AF et al., 2024; Jackson et al., 2019; Laws et al., 2019; Luckett T et al., 2022; Nuti S et al., 2017; Vaitkeviciene et al., 2023; van der Sar et al., 2021; Zeh S et al., 2019) | Care coordination; interprofessional collaboration; integrated and continuous care; information continuity. | 18 |
| **Overall satisfaction** (Ahern et al., 2019; Arnold et al., 2022; Cowell et al., 2024; Goldthorpe J et al., 2018; F. Graham et al., 2021; Huhtanen et al., 2025; Humphries et al., 2021; Krist et al., 2008; Laws et al., 2019; C. Lin et al., 2022; Luckett T et al., 2022; Nuti S et al., 2017; Vaitkeviciene et al., 2023; Wagner et al., 2024; Williams et al., 2018) | Overall satisfaction with care; willingness to recommend and reuse healthcare services. | 16 |
| **Cultural safety** (Bajgain et al., 2020; Frahsa et al., 2020; Fu et al., 2023; Howard AF et al., 2024; Luckett T et al., 2022; Olding et al., 2018) | Cultural responsiveness and safety; respect for cultural values and preferences; cultural and language barriers; inclusion of social and cultural diversity. | 12 |
| **Safety of care** (Abelsen et al., 2023; Bryant et al., 2023; F. Graham et al., 2021; Hopstaken et al., 2021; Luckett T et al., 2022; Montgomery-Taylor S et al., 2016; Pourmarzi et al., 2019; Price et al., 2022; Zeh S et al., 2019; Zigman Suchsland ML et al., 2019) | Perceived safety of care; clinical and environmental safety; service reliability. | 11 |
| **Symptom management** (Ahern et al., 2019; Baker et al., 2015; Bryant et al., 2023; Caneiras C et al., 2022; Luckett T et al., 2022; Savla et al., 2025; Vaitkeviciene et al., 2023; Walker et al., 2017; White TM et al., 2024) | Symptom and pain management; symptom experience; disease control. | 11 |
| **Social support** (Bryant et al., 2023; Frahsa et al., 2020; F. Graham et al., 2021; J. Graham et al., 2007; Jackson et al., 2019; Lamprell K et al., 2023; Vaitkeviciene et al., 2023; van der Sar et al., 2021) | Peer support; group belonging; shared experiences; interpersonal learning. | 11 |
| **Financial access to services** (Ahern et al., 2019; Bajgain et al., 2020; Frahsa et al., 2020; Fu et al., 2023; F. Graham et al., 2021; Tou et al., 2022) | Financial burden; insurance coverage; transportation; service accessibility; social determinants of access to care. | 9 |
| **Physical environment** (Giannopoulos E et al., 2024; Luckett T et al., 2022; McErlane F et al., 2018; Rodríguez-Fuertes et al., 2023; Vaitkeviciene et al., 2023) | Care environment; physical comfort; cleanliness and order. | 8 |
| **Impact on daily life** (Ahern et al., 2019; Baker et al., 2015; Caneiras C et al., 2022; Jackson et al., 2019; Tacchini-Jacquier N et al., 2024; Walker et al., 2017) | Impact on daily functioning; work participation; quality of life. | 7 |
| **Caregiver and family involvement** (Caneiras C et al., 2022; Frahsa et al., 2020; Giannopoulos E et al., 2024; Jackson et al., 2019; Vaitkeviciene et al., 2023; Zeh S et al., 2019) | Caregiver and family involvement; family support. | 6 |
| **Patient preferences** (Bajgain et al., 2020; Fu et al., 2023; F. Graham et al., 2021; Laws et al., 2019; Luckett T et al., 2022) | Care preferences; preferred care delivery; treatment preferences. | 5 |
| **Privacy** (Fu et al., 2023; F. Graham et al., 2021; Luckett T et al., 2022) | Privacy; confidentiality of health information; respect for patient dignity. | 4 |
| **Diagnostic testing experience** (Zigman Suchsland ML et al., 2019) | Physical discomfort; procedure-related discomfort; diagnostic test experience. | 2 |
| **Care needs** (Giannopoulos E et al., 2024; Olding et al., 2018) | Unmet healthcare needs; respect for patients' values and needs. | 2 |
| **Contextual appropriateness of services** (Olding et al., 2018) | Patient experience measurement; use of patient experiences for quality assessment. | 1 |
